# High energy-density and reversibility of iron fluoride cathode enabled via an intercalation-extrusion reaction

**DOI:** 10.1038/s41467-018-04476-2

**Published:** 2018-06-13

**Authors:** Xiulin Fan, Enyuan Hu, Xiao Ji, Yizhou Zhu, Fudong Han, Sooyeon Hwang, Jue Liu, Seongmin Bak, Zhaohui Ma, Tao Gao, Sz-Chian Liou, Jianming Bai, Xiao-Qing Yang, Yifei Mo, Kang Xu, Dong Su, Chunsheng Wang

**Affiliations:** 1 Department of Chemical and Biomolecular Engineering, University of Maryland, College Park, MD 20742 USA; 2 Brookhaven National Laboratory, Upton, NY 11973 USA; 3 Department of Materials Science and Engineering, University of Maryland, College Park, MD 20742 USA; 4 Chemical and Engineering Materials Division, Oak Ridge National Laboratory, Oak Ridge, TN 37831 USA; 5 Maryland Nanocenter, University of Maryland, College Park, MD 20742 USA; 6 Electrochemistry Branch, Power and Energy Division Sensor and Electron Devices Directorate, U.S. Army Research Laboratory, Adelphi, MD 20783 USA

## Abstract

Iron fluoride, an intercalation-conversion cathode for lithium ion batteries, promises a high theoretical energy density of 1922 Wh kg^–1^_._ However, poor electrochemical reversibility due to repeated breaking/reformation of metal fluoride bonds poses a grand challenge for its practical application. Here we report that both a high reversibility over 1000 cycles and a high capacity of 420 mAh g^−1^ can be realized by concerted doping of cobalt and oxygen into iron fluoride. In the doped nanorods, an energy density of ~1000 Wh kg^−^^1^ with a decay rate of 0.03% per cycle is achieved. The anion’s and cation’s co-substitutions thermodynamically reduce conversion reaction potential and shift the reaction from less-reversible intercalation-conversion reaction in iron fluoride to a highly reversible intercalation-extrusion reaction in doped material. The co-substitution strategy to tune the thermodynamic features of the reactions could be extended to other high energy conversion materials for improved performance.

## Abstract (web summary)

Poor electrochemical reversibility of the conversion-type cathode materials remains an important challenge for their practical applications. Here, the authors report a highly reversible fluoride cathode material with low hysteresis through concerted doping of cobalt and oxygen into iron fluoride.

## Introduction

Lithium ion batteries (LIBs) have dominated portable electronics, and are penetrating the markets of electric vehicles. Current LIB cathodes such as LiCoO_2_, LiFePO_4_, or LiNi_*x*_Mn_*y*_Co_1 – *x* – *y*_O_2_ are exclusively based on intercalation mechanism, which involves topotactic intercalation/deintercalation of Li^+^ in a host lattice. However, these cathodes have specific capacities ranged only from 140 to 200 mAh g^−1^, which limit their energy densities^1,2^. Alternatively, certain metallic oxide, sulfide, or halide compounds can experience conversion reactions with Li^+^ by accepting multiple electrons per formula, delivering much higher capacities. These conversion reaction compounds, represented as MX_*y*_ (M = transition metals, X = N, F, O, S, etc.), are converted to a nanocomposite containing reduced M^0^ and Li_*z*_X after discharge (Eq. 1)^3^:

1 $${{\rm{M}}}^{y+}{{{\rm{X}}}^{z-}}_{y/z}+y{{\rm{Li}}}^{+}+y{\mathrm{e}}^{-}{\longleftrightarrow} {{\rm{M}}}^{0}+y/z{{\rm{Li}}}_{z}{\rm{X}}$$

Among them, iron trifluoride (FeF_3_), cheap and environmentally friendly, appears to be unique as a cathode candidate with a total theoretical energy density of 1922 Wh kg^−1^ and a relatively high working potential due to its ionic nature^4^. Its lithiation process goes through two successive intercalation and conversion reactions. In the intercalation reaction at ~3.0 V, FeF_3_ is lithiated into LiFeF_3_ providing a capacity of 237 mAh g^−1^. With further lithiation to below 2.0 V, the LiFeF_3_ will decompose into Fe and LiF through a conversion reaction providing another 475 mAh g^−1^ (Eq. 2)^5^.

2 $${\rm FeF}_{3}{{{{\rm Li}^{+}+{\rm e}^{-}\,{\rm intercalation}}\atop{\longleftrightarrow }} \atop {237\,{\rm{mAh}}\,{{\rm{g}}}^{-1}}}\begin{array}{c}{\rm LiFeF}_{3}\\ ({\rm rutile})\end{array}{{{2{\rm Li}^{+}+2{\rm e}^{-}\,{\rm conversion}}\atop{\longleftrightarrow }}\atop {475\,{\rm{mAh}}\,{{\rm{g}}}^{-1}}}3{\rm LiF}+{\rm Fe}$$

Although extensive researches have been conducted, only limited performance improvement was achieved due to several intrinsic issues of conversion reaction: (1) a severe voltage hysteresis (~1.3 V) between the lithiation and delithiation processes, thus a low round-trip energy efficiency of <60% was always observed^6–8^, due to the slow phase separation and repeated breaking/reforming of Fe–F bonds in each conversion reaction cycle^9,10^; (2) sluggish conversion reaction kinetics, which is caused by both poor electronic conduction, low ion diffusivity in FeF_3_^8^, and the slow conversion phase transition, leads to extremely poor rate capabilities^7,11–13^; (3) the aggregation and continuous coarsening of Fe nanoparticles^13,14^ during repeated conversion reaction cycles, and sustained reactions of Fe with electrolytes^15,16^, result in the rapid capacity decay during cycling^4,16–30^. Partial substitution of fluorine with oxygen (FeO_*x*_F_2−*x*_ (0.4 < *x* < 0.7)) enable the formation of an intermediate rocksalt phase through a reversible extrusion reaction before the conversion reaction of rocksalt phase. During cell discharge, the incoming Li^+^ enter the parent phase and prompt the precipitation of metal and LiF phase. On recharge, these precipitated phases go into the parent phase with the Li^+^ pumped out. Here the parent phase is the defected rocksalt phase. The lithiation of FeO_*x*_F_2−*x*_ experiences intercalation-extrusion-conversion reaction pathway. Since the less-reversible conversion reaction was partially replaced by a highly reversible extrusion reaction in FeO_*x*_F_2−*x*_ (0.4 < *x* *<* 0.7), electrochemical performance was significantly enhanced (Eq. 3)^6,31–37^. However, the reaction kinetics, cycle life, and round-trip efficiency of FeO_*x*_F_2−*x*_ (0.4 < *x* *<* 0.7) still are far less satisfactory when compared to intercalation cathodes due to the existence of less-reversible conversion reaction.

3 $$\begin{array}{c}\begin{array}{c}{\mathrm{FeOF}}\\ ({\mathrm{rutile}})\end{array} \,\mathop{\longleftrightarrow }\limits^{{\mathrm{L}}{{\mathrm{i}}}^{+}+{\rm e}^{-}{\mathrm{intercalation}}}\begin{array}{c}{\mathrm{LiFeOF}}\\ ({\mathrm{rutile}})\end{array}\\ \,\mathop{\longleftrightarrow }\limits^{(x+y-1){\mathrm{L}}{{\mathrm{i}}}^{+}+(x+y-1){\rm e}^{-}{\mathrm{Extrusion}}\,{\mathrm{I}}}\begin{array}{c}{\mathrm{L}}{{\mathrm{i}}}_{x}{\mathrm{FeO}}{{\mathrm{F}}}_{1-y}\\ ({\mathrm{rocksalt}})\end{array}+y{\mathrm{LiF}}\\ \,\mathop{\longleftrightarrow }\limits^{(1.5-{{x}}-{{y}}){\mathrm{L}}{{\mathrm{i}}}^{+}+(1.5-{{x}}-{{y}}){{\rm{e}}}^{-}{\rm{Extrusion}}\,{\rm{II}}}0.5\begin{array}{c}{\mathrm{LiFe}}{{\mathrm{O}}}_{2}\\ ({\mathrm{rocksalt}})\end{array}+{\mathrm{LiF}}+0.5{\mathrm{Fe}}\\ \,\mathop{\longleftrightarrow }\limits^{1.5{\mathrm{L}}{{\mathrm{i}}}^{+}+1.5{{\rm{e}}}^{-}{\mathrm{Conversion}}}{\mathrm{Fe}}+{\mathrm{LiF}}+{\mathrm{L}}{{\mathrm{i}}}_{2}{\mathrm{O}}\end{array}$$

In recent years, the critical issues of high potential hysteresis (>1 V), poor rate capability, and limited cycling stability of conversion electrodes have been believed to be intrinsic nature of the conversion reaction chemistry, and the hope of using conversion reaction materials in the next-generation lithium batteries waned. However, it will be plausible to suppress the conversion reaction by extending the capacity of highly reversible intercalation-extrusion reaction, thus achieving both high capacity and long cycle life.

In this work, we report that such a high-performance Fe_0.9_Co_0.1_OF cathode with high energy density of ~1000 Wh kg^−1^ and long cycle life of 1000 cycles can be realized by a cost-effective and simple strategy of concerted doping Co/O in FeF_3_. At a charge/discharge current of 500 mA g^−1^, the Fe_0.9_Co_0.1_OF can deliver a capacity of 350 mAh g^−1^ for 1000 cycles. The first-principles calculations and comprehensive characterizations revealed that the less-reversible conversion reaction was entirely prohibited by reducing the potential *f* and the reversibility of the extrusion reaction II was significantly enhanced by formation of non-stoichiometric rocksalt phase with only <5% of Fe(Co) phase in lithiated Fe_0.9_Co_0.1_OF after 100 cycles (Eq. 4), while 50% of Fe was reported in lithiated FeO_*x*_F_2 − *x*_ (0.4 < *x* *<* 0.7)^38^. The thermodynamic capacity reduction due to the elimination of conversion reaction was compensated by kinetic capacity increase due to the enhanced reaction kinetics of extrusion II through formation of defect-rich (especially O vacancies) rocksalt phase. The highly reversible structural transition in the intercalation and extrusion reactions after co-substitution significantly improved performance of Fe_0.9_Co_0.1_OF, which provides an alternative solution to the similar challenges faced by other conversion reaction materials.

4 $$\begin{array}{c}\begin{array}{c}{\mathrm{Fe}}({\mathrm{Co}}){\mathrm{OF}}\\ ({\mathrm{rutile}})\end{array} \,\mathop{\longleftrightarrow }\limits^{{\mathrm{L}}{{\mathrm{i}}}^{+}+{{\mathrm{e}}}^{-}\,{\mathrm{I}}{\mathrm{n}}{\mathrm{t}}{\mathrm{e}}{\mathrm{r}}{\mathrm{c}}{\mathrm{a}}{\mathrm{l}}{\mathrm{a}}{\mathrm{t}}{\mathrm{i}}{\mathrm{o}}{\mathrm{n}}}\begin{array}{c}{\mathrm{LiFe}}({\mathrm{Co}}){\mathrm{OF}}\\ ({\mathrm{rutile}})\end{array}\\ \,\mathop{\longleftrightarrow }\limits^{({{x}}+{{y}}-1){\mathrm{L}}{{\mathrm{i}}}^{+}+({{x}}+{{y}}-1){{{\rm e}}}^{-}\,{\mathrm{Extrusion}}\,{\mathrm{I}}}\begin{array}{c}{\mathrm{L}}{{\mathrm{i}}}_{{{x}}}{\mathrm{Fe}}({\mathrm{Co}}){\mathrm{O}}{{\mathrm{F}}}_{1-{{y}}}\\ ({\mathrm{rocksalt}})\end{array}+{{y{\mathrm{LiF}}}}\\ \,\mathop{\longleftrightarrow }\limits^{(1.5-{{x}}-{{y}}){\mathrm{L}}{{\mathrm{i}}}^{+}+(1.5-{{x}}-{{y}}){{{\rm e}}}^{-}\,{\mathrm{Extrusion}}\,{\mathrm{II}}}\frac{1}{2-{{z}}}\begin{array}{c}{\mathrm{L}}{{\mathrm{i}}}_{1-\frac{{{z}}}{2}}{\mathrm{Fe}}({\mathrm{Co}}){{\mathrm{O}}}_{2-{{z}}}\\ ({\mathrm{defected}}\,{\mathrm{rocksalt}})\end{array}\\ \,\,\,\,+{\mathrm{LiF}}+(1-\frac{1}{2-{{z}}}){\mathrm{Fe}}({\mathrm{Co}})\end{array}$$

## Results

### Synthesis and characterization

**Fig. 1:**
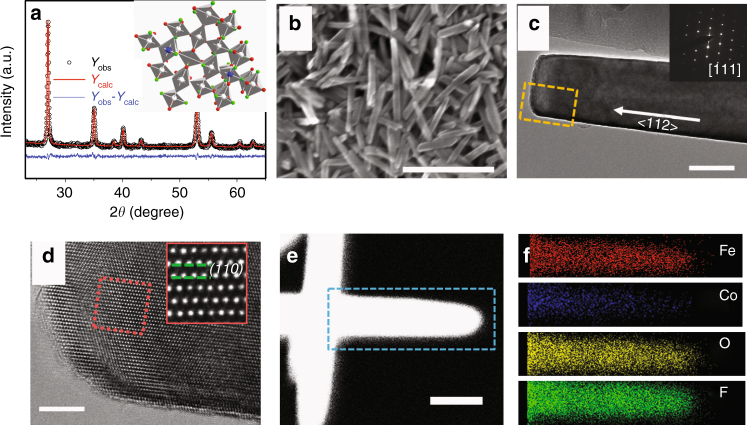
Structural and morphological characterization of Fe_0.9_Co_0.1_OF. **a** Rietveld refinement showing data points (*Y*_obs_, black circles), calculated profile (*Y*_calc_, red line), and difference profile (blue line), as indicated. Refined parameters: *a* = 4.68(6) Å, *c* = 3.06(6) Å, which are slightly larger than those of FeOF (*a* = 4.647 Å, *c* = 3.048 Å). **b** SEM images of the Fe_0.9_Co_0.1_OF nanorods; **c**, **d** TEM and HRTEM images of Fe_0.9_Co_0.1_OF; **e**, **f** STEM-HAADF image of Fe_0.9_Co_0.1_OF nanorod and STEM-EDS elemental mapping of Fe, Co, O, and F. Inset of **a**: the crystal structure of Fe_0.9_Co_0.1_OF. The insets of **c**: corresponding SAED pattern. The insets of **d**: enlargement HRTEM image of the HRTEM image. The rectangle in **e** denotes the EDS mapping area in **f**. Scale bar in **b**–**e** is 400, 50, 5, and 50 nm, respectively

Fe_0.9_Co_0.1_OF and FeOF were synthesized using a solvothermal method, while FeF_3_ was prepared by ball milling the commercial FeF_3_ for 6 h (Supplementary Fig. 1). FeF_3_, FeOF, and Fe_0.9_Co_0.1_OF powders show distinct colors: FeF_3_ is light green; FeOF appears brown; while Fe_0.9_Co_0.1_OF is dark brown (Supplementary Fig. 2). This darkening trend indicates the change of the bandgap after doping, which is verified by the density functional theory (DFT) calculations (Supplementary Fig. 3). The crystal structures of FeOF and Fe_0.9_Co_0.1_OF were examined using X-ray diffraction (XRD), scanning electron microscopy (SEM), transmission electron microscopy (TEM) and high-resolution (HR) TEM, respectively. Figure 1a shows the XRD pattern of Fe_0.9_Co_0.1_OF, which has the same tetragonal rutile structure as FeOF with a slightly increased unit cell size (Supplementary Fig. 4). SEM (Fig. 1b) image reveals a uniform nanorod morphology of Fe_0.9_Co_0.1_OF with a diameter of about 40–50 nm and length of 300–400 nm. Under the same synthesis conditions, the FeOF particle is much larger (Supplementary Fig. 5). However, by changing the solvent and the temperature, FeOF nanorods with the same size as Fe_0.9_Co_0.1_OF was obtained (Supplementary Fig. 6). A representative TEM image (Fig. 1c) and corresponding selected area electron diffraction (SAED) pattern at inset show the growth direction as <112>. HRTEM image (Fig. 1d) in Fig. 1c further reveal the lattice spacing of 0.33 nm ((110) plane) for one-dimensional Fe_0.9_Co_0.1_OF nanorods. The elemental distribution of nanorod was investigated with an energy-dispersive X-ray spectroscopy (EDS) technique in scanning TEM (STEM) mode. A high-angle annular dark-field (HAADF)-STEM image (Fig. 1e) and STEM-EDS elemental mappings (Fig. 1f) show that all four elements (Fe, Co, O, and F) are uniformly distributed throughout the nanorod, which is consistent with the cross-sectional STEM-EDS line profile of Fe_0.9_Co_0.1_OF nanorod (Supplementary Fig. 7). EDS results also show that the atomic ratio of the O:F in Fe_0.9_Co_0.1_OF and FeOF is almost the same and close to 1 (Supplementary Fig. 8).

### Electrochemical performance

**Fig. 2:**
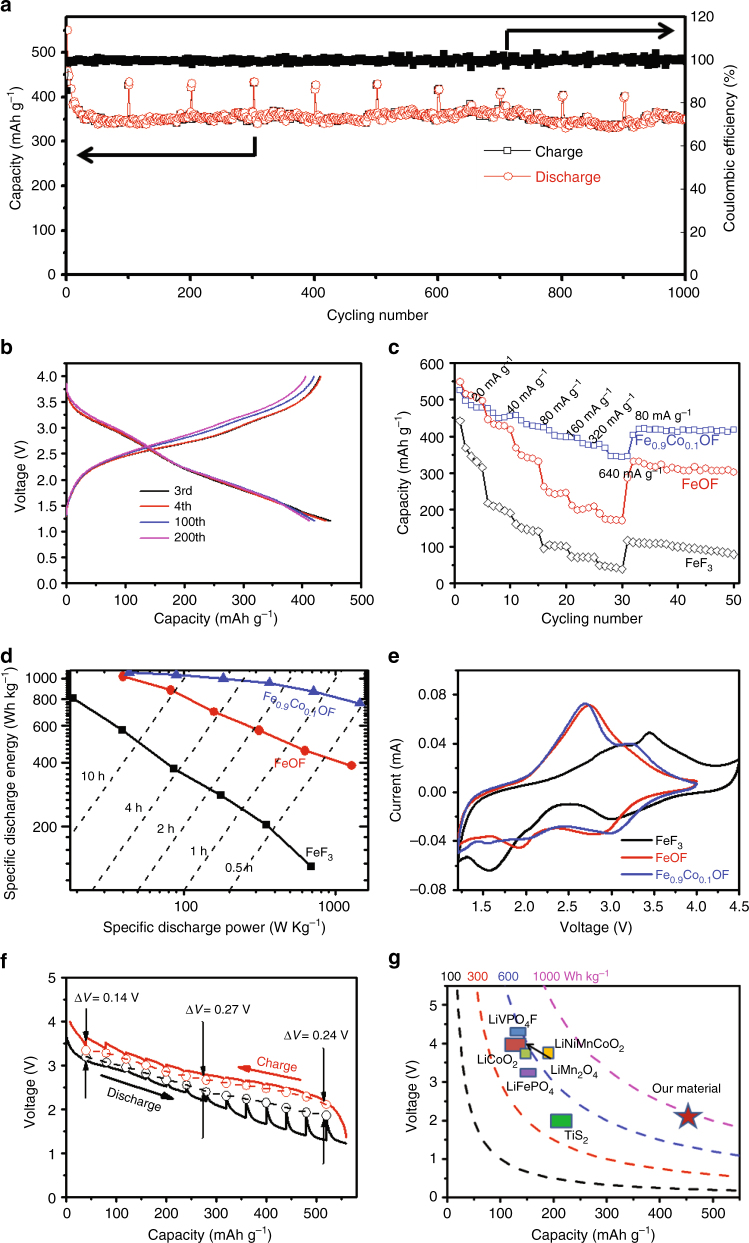
Electrochemical performance of the Fe_0.9_Co_0.1_OF cathode. **a** Cycling performance for Fe_0.9_Co_0.1_OF cathode with a current of 500 mA g^−1^, every 100 cycles, the current changed to 70 mA g^−1^ and cycled two cycles; **b** representative discharge/charge curves for Fe_0.9_Co_0.1_OF at a current of 70 mA g^−1^ in the potential range of 1.2–4.0 V; **c** rate capability for Fe_0.9_Co_0.1_OF, FeOF, and FeF_3_ cathode materials, respectively; **d** the Ragone plot of FeF_3_, FeOF, and Fe_0.9_Co_0.1_OF (based on active mass); **e** CV profiles for the three materials (FeF_3_, FeOF, and Fe_0.9_Co_0.1_OF) with a scanning rate of 0.1 mV s^−1^. **f** Galvanostatic intermittent titration technique (GITT) measurement of Fe_0.9_Co_0.1_OF (40 mA g^−1^ for 1 h followed by a 20 h rest); **g** capacity, voltage, and energy density of Fe_0.9_Co_0.1_OF (discharge energy density is calculated based on discharge current of 40 mA g^−1^) as compared with the intercalation cathodes

An unexpected high cycling stability is achieved for Fe_0.9_Co_0.1_OF nanorods. Figure 2a displays a stable capacity of 350 mAh g^−1^ at a high current of 500 mA g^−1^ for 1000 cycles, while a higher capacity of 420 mAh g^−1^ can be achieved at a low current of 70 mA g^−1^ for over 330 cycles (Supplementary Fig. 9). The capacity’s decay is about 0.005% at a high current of 500 mA g^−1^, which is unprecedented for any conversion reaction materials and comparable to that of intercalation cathodes. The superior cycling stability is also evidenced by the almost identical discharge profiles at the 200th to the curves at the 100th and the 3rd cycles (Fig. 2b). The charge/discharge profiles of Fe_0.9_Co_0.1_OF cathodes at 70 mA g^−1^ (Fig. 2b) are characterized by a declining discharge pseudo-plateau at 3.0 V followed by an extrusion reaction sloping line. In contrast, the FeF_3_ has a similar initial capacity of ~400 mAh g^−1^ at the same current of 70 mA g^−1^ but it suffers a rapid capacity loss to 120 mAh g^−1^ after only 25 cycles (Supplementary Fig. 9), which is in line with the reported performance^4^. The electrochemical performance of FeOF with different nanorod size is shown in Supplementary Fig. 10. Two FeOF with different sizes show a similar initial capacity of 450 mAh g^−1^ that gradually reduced to 300 mAh g^−1^ within 100 cycles at a current of 70 mA g^−1^ (Supplementary Fig. 10).

Fe_0.9_Co_0.1_OF nanorods show much higher rate capability than those of FeF_3_ and FeOF (Fig. 2c, and Supplementary Fig. 11)^39,40^. Fe_0.9_Co_0.1_OF delivers an enhanced capacity of ~440 mAh g^−1^ at 80 mA g^−1^. When the current is increased to 160, 320 and 640 mA g^−1^, Fe_0.9_Co_0.1_OF still retains a reversible capacity of ~400, ~380, and ~340 mAh g^−1^ (Fig. 2c and Supplementary Fig. 11), respectively, whereas FeOF shows only 300, 250, and 200 mAh g^−1^ at the same rates, and FeF_3_ only possess a reversible capacity of ~60 mAh g^−1^ at 320 mA g^−1^. Figure 2d shows the Ragone plot of FeF_3_, FeOF, and Fe_0.9_Co_0.1_OF (based on active mass). At a lower specific power (a low discharge rate), FeOF or Fe_0.9_Co_0.1_OF show a similar energy density of 1000 Wh kg^−1^. However, Fe_0.9_Co_0.1_OF shows the highest rate capability across the entire rate range: at 640 mA g^−1^, the energy density of Fe_0.9_Co_0.1_OF is twice that of FeOF, or six times higher than that of FeF_3_, yielding the highest energy density ever reported for iron fluoride conversion reaction cathode materials at similar rates.

The electrochemical behavior of Fe_0.9_Co_0.1_OF was characterized using cyclic voltammetry (CV) and galvanostatic intermittent titration technique (GITT). Figure 2e compares the CV scans of Fe_0.9_Co_0.1_OF, FeOF, and FeF_3_. All three fluorides experiences a highly reversible intercalation reaction peaks at around 3.0 V. However, the followed conversion reaction peak at 1.6 V for FeF_3_ shifted to a high potential of 1.9 V in FeOF and the peak current was significantly reduced. The conversion reaction peak completely disappeared in Fe_0.9_Co_0.1_OF, indicating that the conversion reaction was replaced by two extrusion reactions. During delithiation, two corresponding anodic peaks can be observed. The quasi-thermodynamic potential hysteresis of Fe_0.9_Co_0.1_OF (Fig. 2f), FeOF, and FeF_3_ (Supplementary Fig. 12) were measured using GITT. The hollow circles represent quasi-equilibrium potentials after fully relaxation at open circuit for 20 h, which approximates thermodynamic values. At 50% state of charge/discharge, this potential hysteresis of Fe_0.9_Co_0.1_OF is only 0.27 V (Fig. 2f), which is substantially lower than 1.1 V for FeF_3_ and 0.57 V for FeOF (Supplementary Fig. 12), respectively, and is even comparable to some intercalation-type cathodes^41^. The over-potential in Fe_0.9_Co_0.1_OF (170 mV) is also significantly smaller than the reaction in FeOF (286 mV) and FeF_3_ (634 mV) (Supplementary Fig. 13), and comparable to the lowest over-potential ever reported in mixed metal fluoride cathode (Cu_*y*_Fe_1 − *y*_F_2_, 150 mV)^16^. The high potential hysteresis (>0.5 V) for conversion fluoride cathodes is attributed to the different conversion reaction pathways during lithiation and delithiation^6,7,20^, which was considered as an intrinsic challenge and cannot be overcome through nanostructure design. We overcome this intrinsic issue by replacing the conversion reaction with two extrusion reactions through Co and O co-substitution in FeF_3_, therefore the over-potential value of Fe_0.9_Co_0.1_OF calculated from the middle points of the charge/discharge capacities is only <1/3 of the FeF_3_ (Supplementary Fig. 14), resulting in an energy round-trip efficiency of >80% (Supplementary Fig. 15).

Non-lithiated Fe_0.9_Co_0.1_OF cathode would require a lithiated anode in a full battery as its electrochemical couple, which is extremely difficult from perspective of practical production. For an ideal cathode material to be used as drop-in replacement by LIB industry, it is desirable to exist with built-in lithium source. Hence we pre-lithiated Fe_0.9_Co_0.1_OF via ball milling with LiH (Supplementary Fig. 16). The charge/discharge curves of pre-lithiated Fe_0.9_Co_0.1_OF (Supplementary Fig. 17) exhibit a higher over-potential than its non-lithiated counterpart in the first cycle. In the subsequent cycles, however, the voltage profiles of pre-lithiated Fe_0.9_Co_0.1_OF (Supplementary Fig. 17) became almost identical to its unlithiated Fe_0.9_Co_0.1_OF (Fig. 2b), with a much smaller voltage hysteresis than the pre-lithiated FeF_3_ reported previously^20,40,42^. The cycling performance for the pre-lithiated Fe_0.9_Co_0.1_OF is also similar to the non-lithiated one (Supplementary Fig. 18). The high capacity combined with a high discharge voltage leads to a discharge energy density of ~1000 Wh kg^−1^ at current of 40 mA g^−1^, which is higher than most intercalation cathode materials (Fig. 2g).

### PDF and ex situ TEM characterization

**Fig. 3:**
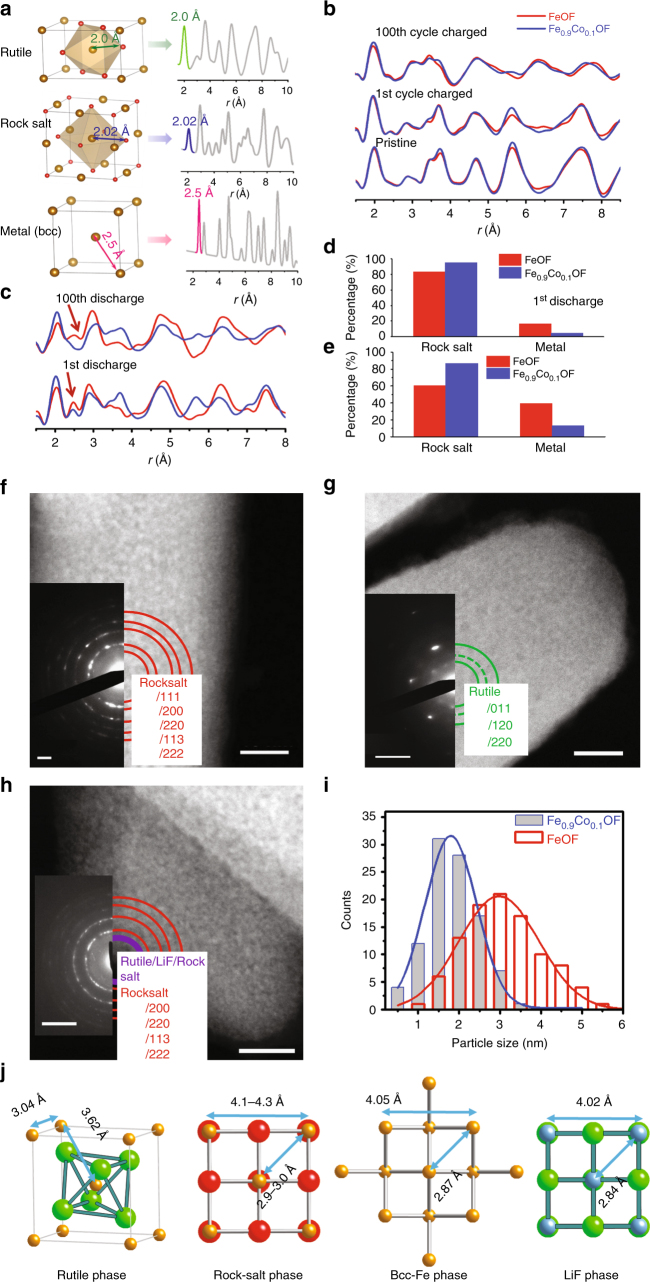
Microstructure evolution during cycling for the Fe_0.9_Co_0.1_OF cathode material. **a** Illustration of the correspondence between PDF peaks and specific atomic pairs. **b**, **c** PDF patterns of Fe_0.9_Co_0.1_OF and FeOF for different charge and discharge states. **d**, **e** The concentration of rocksalt phase, and the metal phase by fitting PDF results after different discharge states. **f**–**h** HRTEM images of Fe_0.9_Co_0.1_OF electrode after discharged to 1.2 V, charged to 4 V, and after 100 cycles discharged, respectively. **i** The histograms of the metal particle size distribution for Fe_0.9_Co_0.1_OF and FeOF electrodes after discharged to 1.2 V. Insets in **f**–**h** are the corresponding SAED patterns. **j** Illustration of the crystal structures of the four structures during cycling. Scale bar in **f**–**h** is 10 nm. Scale bar in the inset of **f**–**h** is 2, 5, and 5 nm^−1^, respectively

The phase evolution of Fe_0.9_Co_0.1_OF during (de)lithiation was analyzed using X-ray pair distribution function (PDF) and compared to that of FeOF. PDF analysis is a powerful tool to study local Fe ordering in FeOF and Fe_0.9_Co_0.1_OF^38,43,44^. Figure 3a displays the PDF patterns of three Fe-containing phases (rutile, rocksalt, and bcc Fe metal), and the corresponding atomic structures. Figure 3b shows the experimental PDF patterns of FeOF and Fe_0.9_Co_0.1_OF at pristine state, charged state after the 1st cycle, and charged state after the 100th cycle. Apparently, all PDF patterns at charged states are similar to that at the pristine, indicating reversible rutile phase formation at charged states in both FeOF and Fe_0.9_Co_0.1_OF. In contrast, significant differences in PDF patterns arise at the discharged states (Fig. 3c). In these PDF patterns, the first peak at about 2.0 Å corresponds to the Fe–O and Fe–F correlation in the oxidized Fe phases (rutile and rocksalt), and the second peak at ~2.5 Å as indicated by the red arrows in Fig. 3c corresponds to the nearest Fe–Fe pairs in metallic phase^38^. The PDF pattern of the discharged FeOF (1st cycle) can be fitted well using rocksalt and metal phases (Supplementary Fig. 19), which contains about 60.6% of rocksalt phase and 39.4% of Fe (Fig. 3d). The metallic Fe (39.4%) in FeOF is lower (50%) than that in oxygen-less FeO_*x*_F_2 − *x*_ (0.4 < *x* < 0.7) materials^38^. In discharged Fe_0.9_Co_0.1_OF, the portion of metallic phase was further reduced to only about 13.1% while the rocksalt phase increases to 86.9% (Fig. 3d and Supplementary Fig. 19). After 100 cycles, the characteristic peak of metal in Fe_0.9_Co_0.1_OF becomes a broadened shoulder (Fig. 3c). Fitting results indicate that the content of metallic Fe(Co) phase was significantly reduced to about 5% while the rocksalt phase dramatically increased to 95% (Fig. 3e). According to Wiaderek et al.^38^, the lithiation of FeOF first goes through Li intercalation followed by phase changes from rutile to rocksalt with the extrusions of Fe and LiF, and then the rocksalt phase completely decomposes into Li_2_O and Fe. The PDF results suggest that the reaction natures of Fe_0.9_Co_0.1_OF and FeOF are different: for Fe_0.9_Co_0.1_OF, conversion reaction is suppressed and the reversibility of extrusion reaction II is enhanced by suppressing the formation metallic phase through co-substitution. It indicates that the main reaction in Fe_0.9_Co_0.1_OF is an intercalation-extrusion reaction between rutile and rocksalt phase (Eq. 4). The mechanism for structure enhanced electrochemical performance will be discussed later.

The morphology and phase upon cycling were investigated using HRTEM, STEM, and SAED (Supplementary Fig. 20 and Fig. 3f–h). When Fe_0.9_Co_0.1_OF was discharged to 1.2 V, bright contrast of small nanoparticles from STEM imaging was observed and its SAED pattern indicates that the main phase of discharged Fe_0.9_Co_0.1_OF is rocksalt (Fig. 3f). After being charged back to 4 V, rocksalt phase changed back to the rutile phase (Fig. 3g). In the discharged state after 100 cycles (Fig. 3h), the rocksalt phase still dominates Fe_0.9_Co_0.1_OF, which is consistent to the PDF results and previous reports^38,45^. In these SAEDs, we observed the spotty-like diffraction patterns indicating large sizes of rutile/rocksalt grains, whereas in HAADF-STEM images, the small bright nanoparticles and its corresponding broaden rings in diffraction patterns^33,46^ are attributed to the metallic nanoparticles. Considering HRTEM images and their fast Fourier transform (Supplementary Fig. 20), we conclude that the rocksalt phase accounts for the main part of discharged Fe_0.9_Co_0.1_OF with small amount of metallic Fe(Co). In addition, the Co-doping in FeOF reduces the size of metallic nanoparticles in discharged Fe_0.9_Co_0.1_OF. Figure 3i compares the size distributions of nanoparticles formed in FeOF and Fe_0.9_Co_0.1_OF after second discharging to 1.2 V, where the Fe/Co particle size from Fe_0.9_Co_0.1_OF are between 0.5 and 3.5 nm with an average size of 1.8 nm, while the Fe particles in dicharged FeOF widely distributes between 2 and 5.5 nm with average size of 3 nm (Supplementary Fig. 21). In comparison, those decomposed from FeF_3_ are around 5 nm^13^. Figure 3j show that the rocksalt phase (Li_*x*_FeO_2 − y_), Fe metal, and LiF have similar lattice constants. The cubic-on-cubic coherence of their lattice can help to form a composite to maintain the overall shape of nanorods, thus maintaining morphology and structure integration during cycling.

### In situ TEM investigation of structural evolution

**Fig. 4:**
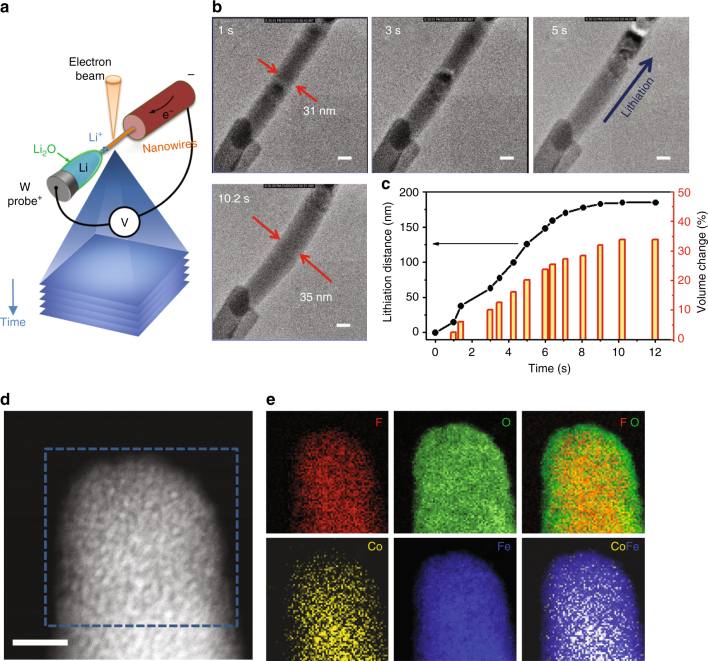
Structure conversion of Fe_0.9_Co_0.1_OF nanorod monitored in real time and the elemental composition distribution. **a** Schematic illustration of the electrochemical setup for in situ TEM test. **b** In situ HRTEM image of Fe_0.9_Co_0.1_OF during dynamic lithiation at 1.2 V for different time. **c** The lithiation distance and the volume change as function of time. **d** ADF-STEM image and **e** corresponding Fe, Co, O, and F elemental distributions after the first lithiation obtained by STEM-EELS technique. Scale bar in **b** and **d** is 20 and 10 nm, respectively

The structural evolution of Fe_0.9_Co_0.1_OF during cycling was further investigated by in situ TEM technique with an experimental setup schematically illustrated in Fig. 4a. The time-lapse images and movie for lithiation are shown in Fig. 4b, and Supplementary Movie 1, and those for delithiation are shown in Supplementary Fig. 22 and Supplementary Movie 2, respectively. A clear contrast change was observed during Li insertion reaction^46,47^, and the reaction front moves rapidly across the nanorod (from the lower left to upper right). Compared with the particle-to-particle diffusion in FeF_3_^47^, Fe_0.9_Co_0.1_OF exhibits a much faster reaction rate without any obvious variations on surface and in bulk. Interestingly, the lithiation of a 200 nm Fe_0.9_Co_0.1_OF nanorod occurs within only ~10 s. In comparison, for FeF_3_ nanoparticles with much smaller size of ~10 nm, it takes at least a few minutes for full lithiation^47^. Figure 4c compares the lithiation distance and volume change vs. time. It agrees with the electrochemical performances shown in Fig. 2c, d, and indicates that the reaction kinetics after Co/O co-substitution is largely improved^12,48^. During lithiation of Fe_0.9_Co_0.1_OF, the nanorod volume expands by ~35% with a little bend due to the stress, but no obvious cracks are observed.

The STEM-EDS results have confirmed that Fe and Co elements are homogeneously dispersed in Fe_0.9_Co_0.1_OF nanorods at both pristine (Fig. 1, Supplementary Fig. 7) and charged states (Supplementary Fig. 23). In addition, the chemical distributions of Fe_0.9_Co_0.1_OF upon cycling have been investigated using STEM-electron energy-loss spectroscopy (EELS) mapping and line-scan (Fig. 4d, e and Supplementary Fig. 24). It is found that the surface of lithiated Fe_0.9_Co_0.1_OF is covered with 1.5~2 nm oxygen-rich surface layer (Fig. 4e), which is still observed even after 100 cycles (Supplementary Fig. 24c). In Supplementary Fig. 24c, at the surface (position of 8 nm), the O content is much higher than F content. However, no such layer was observed in FeOF. Instead, nano-sized domains with size of 10–20 nm were observed, where significant element segregations can be detected (Supplementary Fig. 25). We believe that the surface O-rich rocksalt layer has been effectively stabilized by Co-doping, which consequently inhibits the reaction of electrolyte with the nascent metal nanoparticles and the subsequent dissolution of transition metals into electrolytes^33^. This conclusion is evidenced by the elemental analysis using inductively coupled plasma atomic emission spectroscopy (Supplementary Fig. 26), where a much lower transition metal concentration is detected in the electrolytes for Fe_0.9_Co_0.1_OF than that for FeOF after prolonged cycling. It should be noted that this O-rich oxide layer with thickness of ~2 nm will not retard the Li insertion in later cycles according to DFT energy barrier calculations (Supplementary Fig. 27 and Suppementary Note 1). The diffusion barrier in LiFeO_2_ rocksalt phase is calculated to be 0.29 eV, which is similar to that in LiFePO_4_ cathode materials^49^.

### Reaction mechanism of Fe_0.9_Co_0.1_OF

From the structural characterizations using PDF and TEM, we have observed the following changes after doping Co into FeOF: (1) after the first fully lithiation, the amount of metal reduced from 39.4 to 13% while the amount of rocksalt phase significantly increased from 60.6 to ~86.9%. After 100 cycles, the amount of metallic phase in lithiated Fe_0.9_Co_0.1_OF was further reduced to ~5%. (2) The particle size of FeCo_*x*_ in discharged state reduced from 3.0 to 1.8 nm and the FeCo_*x*_ particle size was further reduced during charge/discharge cycles and almost not detectable after 100 cycles. (3) Fe_0.9_Co_0.1_OF possesses a stable oxygen-rich surface layer during long-term cycling.

**Fig. 5:**
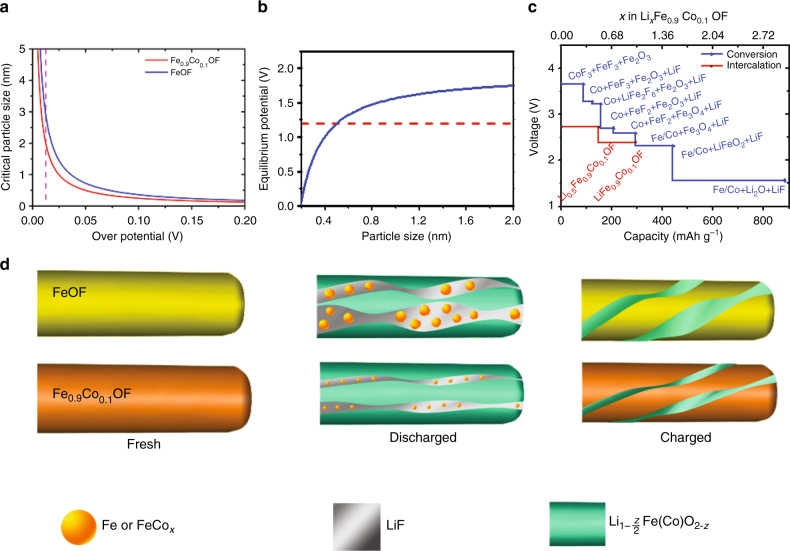
Reaction mechanism for the Fe_0.9_Co_0.1_OF cathode. **a** The relationship between the overpotentials and the metal critical particle sizes during lithiation for FeOF and Fe_0.9_Co_0.1_OF. **b** The relationship between the in situ-formed metal particle size with the equilibrium potential of the conversion reaction. **c** Calculation reaction curves of the Fe_0.9_Co_0.1_OF materials from the DFT intercalation of Li into Fe_0.9_Co_0.1_OF, and the conversion path using the equilibrium phases (assuming the size of metal particles is 1 nm). **d** Illustrations of structural evolution for FeOF and Fe_0.9_Co_0.1_OF during lithiation

First, it should be pointed out that similar to the FeF_3_, the surface energy barrier has minor impact on the Li diffusion in the Fe_0.9_Co_0.1_OF system according to the DFT calculation results (Supplementary Fig. 28, Supplementary Note 2, Supplementary Table 1, and Supplementary Note 3). The surface diffusion barrier (0.88 eV) is lower than the bulk diffusion barrier (1.36 eV) in Fe_0.9_Co_0.1_OF. To decipher the mechanism for reducing the Fe particle size from Co substitution, we calculated the nucleation rate of metallic phase from interface energies during the lithiation (Supplementary Fig. 29), and the formation energy of Fe and FeCo_*x*_ particles using first-principles calculations. These calculations indicate that the formation of FeCo_*x*_ nanoparticles becomes much easier in Fe_0.9_Co_0.1_OF, significantly reducing the critical nucleation size of FeCo_*x*_ particles in Fe_0.9_Co_0.1_OF compared to Fe particles in FeOF (Fig. 5a). The critical particle sizes of FeCo_*x*_ and Fe at an over-potential of 0.043 V are 1.8 and 2.9 nm, respectively (indicated by the dashed line in Fig. 5a), corresponding to a ratio of 3.4 for the number of particles in Fe_0.9_Co_0.1_OF over FeOF (Eq. 13), which is in consistence with the observed FeCo_*x*_ and Fe sizes (Fig. 3i). The reduced Fe particle size from 3.0 nm in FeOF to 1.8 nm in Fe_0.9_Co_0.1_OF also reduces the thermodynamic potential for conversion reaction from 1.812 to 1.729 V (Fig. 5c). This conversion potential will be further reduced with cycling due to continuous reduction of FeCo_*x*_ metal nanoparticle size and amount during cycles (Fig. 5b), thus conversion reaction in Fe_0.9_Co_0.1_OF is completely suppressed. The reduction of content and size of FeCo_*x*_ metal nanoparticle in Fe_0.9_Co_0.1_OF is also accompanied by formation of non-stoichiometric rocksalt phase, which significantly enhance the reversibility of extrusion II (Eq. 4). This is confirmed by disappearance of conversion peaks in CV for Fe_0.9_Co_0.1_OF (Fig. 2e) and intercalation and extrusion sloping curves in galvanostatic charge/discharge (Fig. 2b).

DFT calculations (Fig. 5c, and Supplementary Fig. 30) show the initial lithiation process an intercalation reaction (red line in Fig. 5c and Supplementary Fig. 30), and then the LiF and small amount of Fe/Co extrude out of the LiFeO_2_ rocksalt phase, which is consistent to the PDF and TEM investigations (Fig. 3). Theoretically, all levels of lithiation should be “conversion phases”, as shown the blue line in Fig. 5c, and Supplementary Fig. 30. However, given that the small energy difference between the intercalation and the conversion reaction, and the large kinetic barriers to the conversion reactions^34^, it is reasonable that the material intercalate lithium before it converts (red line), which is indeed in consistence with the experiment. Considering the PDF analysis of FeOF by Wiaderek et al.^38^, we propose the lithiation of Fe_0.9_Co_0.1_OF will go through a similar process, involving intercalation and two steps of extrusion reactions (Eq. 4).

On the basis of DFT calculations, ex situ/in situ TEM, and PDF analyses, the lithiation/delithiation of Fe_0.9_Co_0.1_OF in the first charge-discharge cycle can be described by Eq. 4. The fully lithiated Fe_0.9_Co_0.1_OF consist of 87% of non-stoichiometric Li_0.576_Fe(Co)O_1.15_ and 13% of Fe(Co) as quantitatively demonstrated by PDF. The extrusion of LiF from the lithiated materials, which is similar to the lithiation reaction in layered material of Cu_2.33_V_4_O_11_^50^, has fast reaction kinetics without metal accumulation due to low content of Fe(Co). After 100 cycles, the extrusion reaction II become more reversible and fast due to continues reduction of Fe(Co) content (~5%) and size. However, due to the highly defected rocksalt phase, similar capacities were achieved in the cycling. Meanwhile, the lattice coherence of discharged compounds of LiF, rocksalt phase, and metallic phase reduces the interfacial energy and help to improve the reversibility of the compound^34^.

A schematic illustration of comparing the (de)lithiation mechanisms of FeOF and Fe_0.9_Co_0.1_OF is shown in Fig. 5d. When FeOF is fully discharged to a potential of 1.2 V, the FeOF experiences four reactions (Li intercalation, extrusion I, extrusion II, and partial conversion reaction) (Eq. 3). The existence of conversion reaction in the lithiated FeOF after being discharged to 1.2 V still limited the cycling stability due to the high irreversibility of conversion reaction. In contrast, the conversion reaction in Fe_0.9_Co_0.1_OF is completely inhibited and the extrusion reaction II becomes more reversible with charge/discharge cycles. As shown in Supplementary Fig. 31 and Supplementary Note 4 and Note 5, the smaller particle size ensures almost half of the atoms to be located at the interface layer for Fe_0.9_Co_0.1_OF after lithiation, thereby facilities the extrusion reaction and limited the diffusion distance for different ions. This pronounced reduction in particle sizes and stable coherent structures between LiF, metallic phase, and rock phase (Fig. 3d), together with the stabilized O-rich rocksalt surface layer (Fig. 4e, Supplementary Fig. 24) ensure a highly homogeneous distribution of various reaction ingredients, which was proposed as a key rationale behind the unprecedented reversibility^51^. During charge (delithiation), LiF and very small amount of FeCo_*x*_ as well as rocksalt phase will convert back to rutile phase. It is worth mentioning that such reaction mechanism does not always apply for all of the fluoride systems. For example, Wang et al.^16^ showed that there was no intercalation reaction in the Cu_*y*_Fe_1 − *y*_F_2_ materials, suggesting that the nature of reaction mechanism depends on transition metal chemistries. Such dependence in fact provides possibility of tuning the reaction route through elemental doping and tailoring the synthesis route, which is exactly the motivation of this work.

## Discussion

In summary, we successfully improved the cycling stability and reaction kinetics of FeF_3_ by a Co and O co-substitution strategy. We found that the co-substituted Fe_0.9_Co_0.1_OF can achieve an energy density as high as 1000 Wh kg^−1^ (420 mAh g^−1^) at 70 mA g^−1^ for 330 cycles, or 350 mAh g^−1^ for 1000 cycles at a high rate of 500 mA g^−1^. In addition, Co and O substitution of FeF_3_ also significantly reduced the potential hysteresis to 0.27 V. In-depth analysis using analytical/in situ TEM techniques, PDF analysis, and first-principles calculations have revealed that the co-substituted anion (O) and cation (Co) in Fe_0.9_Co_0.1_OF reduced the particles size and abundances of metallic Fe(Co), thus suppressed less-reversible conversion reaction and further enhanced the reversibility of extrusion reactions. The highly reversible intercalation-extrusion reaction pathway of Fe_0.9_Co_0.1_OF enhances the cycling stability and reaction kinetics. Therefore, the Fe_0.9_Co_0.1_OF nanorods achieved exceptional cycling performance with the small hysteresis, highest round-trip energy efficiency, and improved kinetics, which can meet the critical requirements of applications for next-generation cathode materials. The effective co-substitution strategy could be applied to resolve similar reversibility issues encountered by other conversion reaction materials, such as oxides and sulfides.

## Methods

### Material synthesis

Fe_0.9_Co_0.1_OF and FeOF were synthesized via solvothermal method. Typically, FeF_3_∙3H_2_O and CoF_3_ were ball milled at a molar ratio of 9:1 for 30 min to reduce the particle size and get a homogeneous reactant mixture. Then, 170 mg of such mixture was dispersed in 1-propanol (75 ml) and stirred vigorously at room temperature for half an hour. The resulting suspension was transferred into a Teflon-lined 100 ml stainless-steel autoclave reactor, and subsequently sealed and heated at 210 °C for 24 h in an oven. For comparison, FeOF was also synthesized by the same procedure using pure FeF_3_∙3H_2_O as the starting material dispersed in butyl-alcohol. FeF_3_ was prepared by ball milling the as-purchased FeF_3_ for 6 h. The pre-lithiation of Fe_0.9_Co_0.1_OF is synthesized by ball milling Fe_0.9_Co_0.1_OF and LiH at a molar ratio of 1:2 for 10 h.

### Material characterizations

Microstructural analyses were performed by using SEM (Hitachi SU-70) and STEM (JEOL 2100 field emission gun TEM, operated at accelerating voltage of 200 kV), respectively. XRD pattern was recorded by Bruker Smart1000 (Bruker AXS Inc., USA) using Cu Kα radiation.

PDF experiments were carried out at X-ray powder diffraction (XPD) beamline (ID28) at the National Synchrotron Light Source II, Brookhaven National Laboratory, USA, with a photon wavelength of 0.185794 Å. A large-area amorphous-silicon-based detector was used to collect data to high values of momentum transfer (*Q*_max_ = 24 Å^−1^). The raw images were integrated using the software FIT2d^52^. PDFgetX3^53^ was used to correct the data for background contributions, Compton scattering, and detector effects, and to Fourier transform the data to generate *G*_(*r*)_, the PDF.

5 $${G}_{(r)}=4\pi r[{\rho }_{(r)}-{\rho }_{0}]=\frac{2}{\pi }{\int _{0}^{\infty }}Q[{S}_{(Q)}-1]\sin (Qr){{\mathrm{d}Q}}$$

Here *ρ*_(*r*)_ is the microscopic pair density, *ρ*_0_ is the average number density, and *Q* is magnitude of the scattering vector. For elastic scattering *Q* = 4*π*sin(*θ*)/*λ* with 2*θ* being the scattering angle and *λ* the wavelength of the radiation used. *S*_(*Q*)_ is the total scattering function. The intensity and position of peaks corresponding to the Fe–Fe/Co bond in metallic phase (∼2.5 Å) were fitted by using Gaussian functions within fityk^54^. Structure models were refined against the PDF data within PDFgui^55^.

### In situ TEM

A nanofactory scanning tunneling microscopy-TEM holder was used in the experiment. The holder is equipped with three-dimensional piezo-manipulator and biasing capability. Fe_0.9_Co_0.1_OF nanorods were attached on a tungsten probe using conducting epoxy and mounted on one side of the holder. On the other side, we mounted another tungsten rod after transferring a small piece of Li on its tip. The Fe_0.9_Co_0.1_OF nanorods and Li metal were then brought into contact inside the TEM. By applying voltage on the working electrode vs. the counter electrode (Li), lithiation and delithiation processes were recorded. The experiment was performed using a JEOL 2010F TEM operating at accelerating voltage of 200 kV. The HAADF-STEM imaging, STEM-EELS, and STEM-EDX were performed with an aberration-corrected Hitachi HD2700C STEM at 200 kV in Brookhaven National Lab. The conversion angle and collection angles for STEM imaging are 22 and 64–341 mrad, respectively. The collection angle of EELS are from 20 to 26 mrad depending on the needs.

### First-principles calculation methods

All DFT calculations were performed using the Vienna Ab initio Simulation Package^56^ within the projector augmented-wave approach^57^, and the Perdew–Burke–Ernzerhof generalized gradient approximation (GGA) functionals^58^ was used. In structures containing Fe (Co) and O or F, a value of *U* = 4.0 eV (3.32 eV) was used for Fe (Co), as pervious DFT studies. Spin-polarized total energy calculations and structure relaxations were performed. Fe and Co atoms were initialized in high-spin ferromagnetic ordering for simplicity. And the parameters of DFT calculations, such as the plane-wave energy cutoff and *k*-point density, were consistent with the parameters used for the Materials Project (MP). The voltage plateaus are obtained using the calculated DFT energies of all relevant compounds in the Li-Fe-O-F space from the MP^59^. The structural matching results were visualized using VESTA^60^.

### Energy of metal nanoparticles

For the reason that GGA does not capture well the energy difference between the localized *d*-states for ionic iron and cobalt in oxide or fluoride and the delocalized states in metallic iron and cobalt. To solve this problem, as proposed by Doe, the experimentally measured reaction enthalpy obtained for MF_2_ + 2Li = M + 2LiF (M = Fe and Co) and the energies for Li, FeF_2_, and LiF are used to determine the energies of metallic Fe and Co^25^. This approach yields cohesive energies of –4.2811 and 4.3974 eV for bulk, metallic Fe and Co, respectively, corresponding to those obtained from experiment (i.e., –4.28 and –4.39 eV).

To evaluate the nanosize effect, the Wulff shapes of Fe and Co are constructed to give the crystal shape under equilibrium conditions. The Wulff shape is constructed by based on the elemental surface energy data form the database developed by Shyue Ping Ong group^61^. The weighted surface energy $\bar{\gamma }$ using this fraction is given by the following equation:

6 $$\bar{\gamma }=\frac{{\sum }_{\{{\mathrm{hkl}}\}}{\gamma }_{{\mathrm{hkl}}}{A}_{{\mathrm{hkl}}}}{{\sum }^{}{A}_{{\mathrm{hkl}}}}$$

where γ_hkl_ is the surface energy obtained from the database for a unique facet existing in the Wulff shape and *A*_hkl_ is the total area of all facets in the {hkl} family in the Wullf shape. Pymatgen is used to generate slabs and calculate weighted surface energy^61^.

The energy of metal nanoparticle can be calculated by the following equation:

7 $${{E}}({\rm{nano}})={{{E}}}^{{\mathrm{Fit}}}({\rm{bulk}})-\bar{\gamma }\frac{3{V}_{{\mathrm{M}}}}{r{N}_{{\mathrm{A}}}}$$

### Over-potential and critical nucleation particle radius

The particle size was calculated by using the same approach as previous reported^62^. For lithiation of FeOF and Fe_0.9_Co_0.1_OF reaction below, over-potential is the driving force for nucleation. As shown in Figs. 2c and 3a, at a low discharge/charge rate, similar capacity (~480 mAh g^−1^) can be obtained for FeOF and Fe_0.9_Co_0.1_OF, this capacity corresponds to 1.63 mol Li per 1 mol active materials (FeOF or Fe_0.9_Co_0.1_OF), with a decent difference of the metal particle size and the metal contents.

8 $$1.63\,{\rm{Li}}+{\rm{FeOF}}\to 0.39\,{\rm{Fe}}+{\rm{LiF}}+{{\rm{Li}}}_{0.63}{{\rm{Fe}}}_{0.61}{\rm{O}}$$

9 $$\begin{array}{c}1.63\,{\rm{Li}}+{{\rm{Fe}}}_{0.9}{{\rm{Co}}}_{0.1}{\rm{OF}}\to 0.13\,{{\rm{Fe}}}_{0.9}{{\rm{Co}}}_{0.1}\\ +{\rm{LiF}}+{{\rm{Li}}}_{0.63}{{\rm{M}}}_{0.87}{\rm{O}}\end{array}$$

By assuming that the particle has a sphere shape, the formation energy Δ*G* of a particle can be written as a function of over-potential Δ*ϕ* and particle size *r*.

10 $$\Delta G(\Delta \phi ,r)=\frac{4}{3}\pi {r}^{3}\cdot \frac{1}{{V}_{{\rm{M}}}}\cdot \frac{1.63}{x}\cdot \Delta \phi \cdot {N}_{{\mathrm{A}}}-\gamma \cdot 4\pi {r}^{2}$$

where *V*_M_ is molar volume of Fe or Fe_0.9_Co_0.1_ particle. *γ* is the interface energy. *x* is the coefficient of Fe (*x* = 0.39) or Fe_0.9_Co_0.1_ (*x* = 0.13) in reaction Eqs. (9) and (10), respectively. *N*_A_ is the Avogadro constant. Specific *x* values are derived based on PDF studies. As mentioned in the manuscript, the PDF pattern of discharged FeOF can be well fitted by a mixture of rocksalt and metal phases, directly yielding the phase fraction of metal phase (for FeOF, *x* = 0.39). For the Co-substituted one, the estimation of phase fraction was made based on the intensity of the characteristic peak of metal at around 2.5 Å (for Fe_0.9_Co_0.1_OF, *x* = 0.13).

The critical nucleus radius is calculated by

11 $$\frac{\partial {\rm{\Delta }}G({\rm{\Delta }}\phi ,r)}{\partial r}=0$$

Therefore we can get the critical nucleus radius r^*^

12 $${r}^{\ast }({\rm{\Delta }}\phi )=\frac{2\gamma \cdot x\cdot {V}_{{\rm{M}}}}{1.63\cdot {\rm{\Delta }}\phi \cdot {N}_{{\mathrm{A}}}}$$

The relationship between critical particle size and over-potential is shown in Fig. 4i.

Assuming that the same amount of metal particle is converted, then the ratio of nucleation number under same over-potential can be calculated as

13 $$\frac{{N}_{{{\rm{Fe}}}_{0.9}{{\rm{Co}}}_{0.1}}(\Delta \phi )}{{N}_{{\rm{Fe}}}(\Delta \phi )}={\left(\frac{1/{r}_{{{\rm{Fe}}}_{0.9}{{\rm{Co}}}_{0.1}}^{\ast }(\Delta \phi )}{1/{r}_{{\rm{Fe}}}^{\ast }(\Delta \phi )}\right)}^{3}$$

### Electrochemical tests

The electrochemical tests were performed using a coin-type half cell (CR 2032). Metallic lithium was used as the negative electrode. To prepare working electrode, the as-synthesized Fe_0.9_Co_0.1_OF (or FeOF, FeF_3_), carbon black, and polyvinylidene fluoride with mass ratio 70:15:15 were mixed into a homogeneous slurry in NMP with pestle and mortar. The slurry mixture was coated onto Al foil and then dried at 100 °C for 12 h under air atmosphere. For the pre-lithiated Fe_0.9_Co_0.1_OF materials, all of the preparing procedures for the electrode were protected by Ar to avoid the possible oxidation of in situ-formed Fe nanoparticles. The electrolyte consists of 1.0 M LiPF_6_ dissolved in mixture of fluoroethylene carbonate, 2,2,2-trifluoroethyl methyl carbonate, and 1,1,2,2-tetrafluoroethyl-2′,2′,3′,3′-tetrafluoropropyl-ether (HFE) at the weight ratio of 20:60:20. The cells were assembled with a polypropylene microporous film (Celgard 3501) as the separator. Electrochemical performance was tested using Arbin battery test station (BT2000, Arbin Instruments, USA). Capacity was calculated on the basis of the mass of active species (Fe_0.9_Co_0.1_OF, FeOF, or FeF_3_). The energy density of the cathode was machine generated on the Arbin Instruments. Cyclic voltammogram scanned at 0.1 mV s^−1^ between 1.2 and 4.5 V was recorded using a CHI 600E electrochemical workstation (CH Instruments Inc., USA).

### Data availability

All relevant data are available from the authors on request.

## Electronic supplementary material

Supplementary Information

Peer Review File

Description of Additional Supplementary Files

Supplementary Movie 1

Supplementary Movie 2
