# Supplementary material for: High energy-density and reversibility of iron fluoride cathode enabled via an intercalation-extrusion reaction
Source: Nat Commun. 2018 Jun 13;9:2324. doi: 10.1038/s41467-018-04476-2 (PMC5998086; doi:10.1038/s41467-018-04476-2)
Supplement: Supplementary file 1 — Supplementary Information [file 41467_2018_4476_MOESM1_ESM.pdf]

Supplementary information

High Energy-Density and Reversibility of Iron Fluoride Cathode Enabled Via an Intercalation-Extrusion Reaction

Fan. et al.

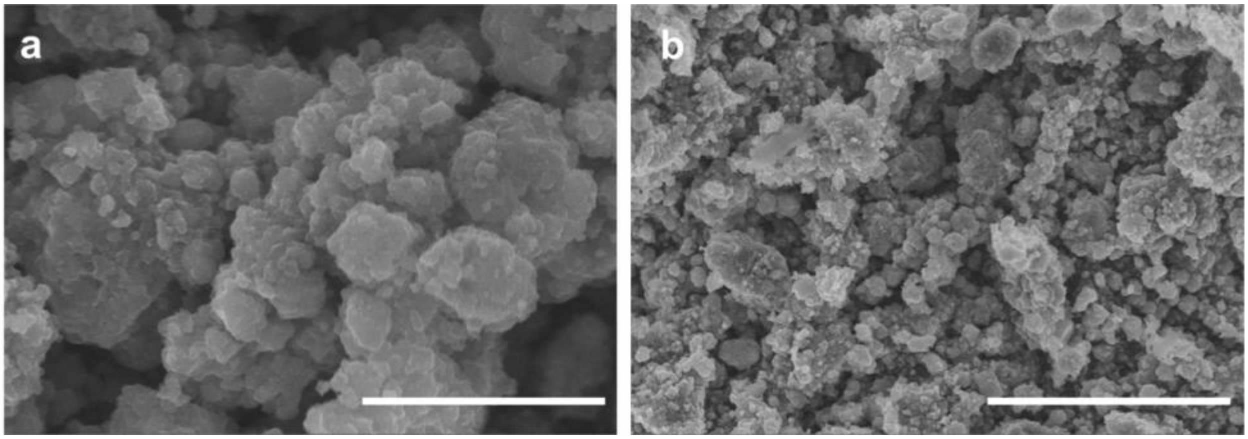

Supplementary Figure 1 SEM images for the FeF_3 after 6 h ball milling. Scale bar in a and b is 2 µm and 10 µm, respectively.

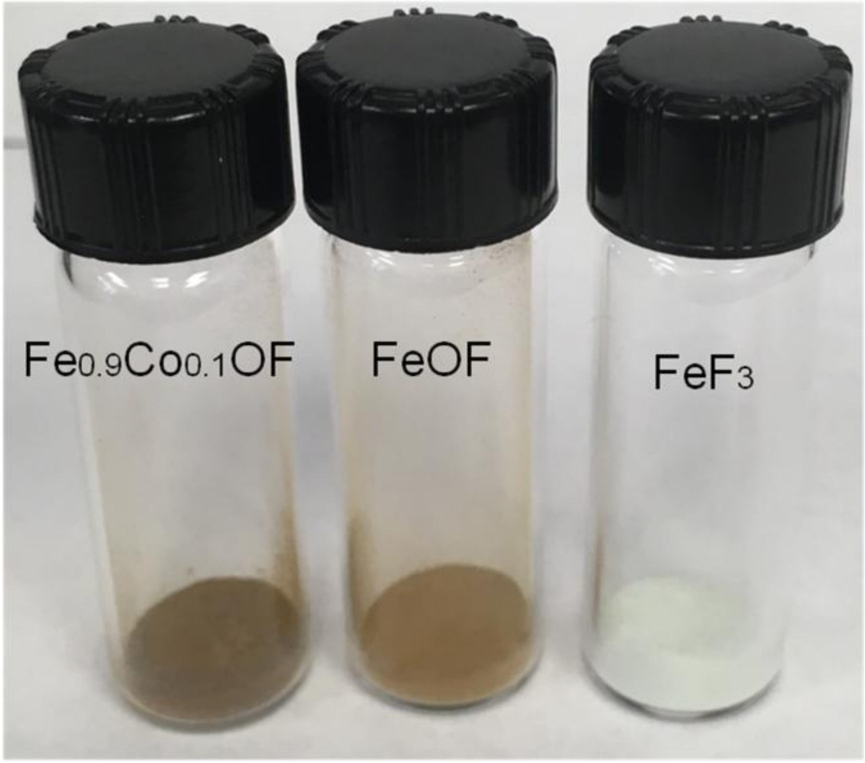

Supplementary Figure 2 Optical image of the as-synthesized $Fe_{0.9}Co_{0.1}OF$, FeOF nanorod material and FeF_3. The colors of FeF_3, FeOF and $Fe_{0.9}Co_{0.1}OF$ are light blue, yellow, and dark brown, respectively.

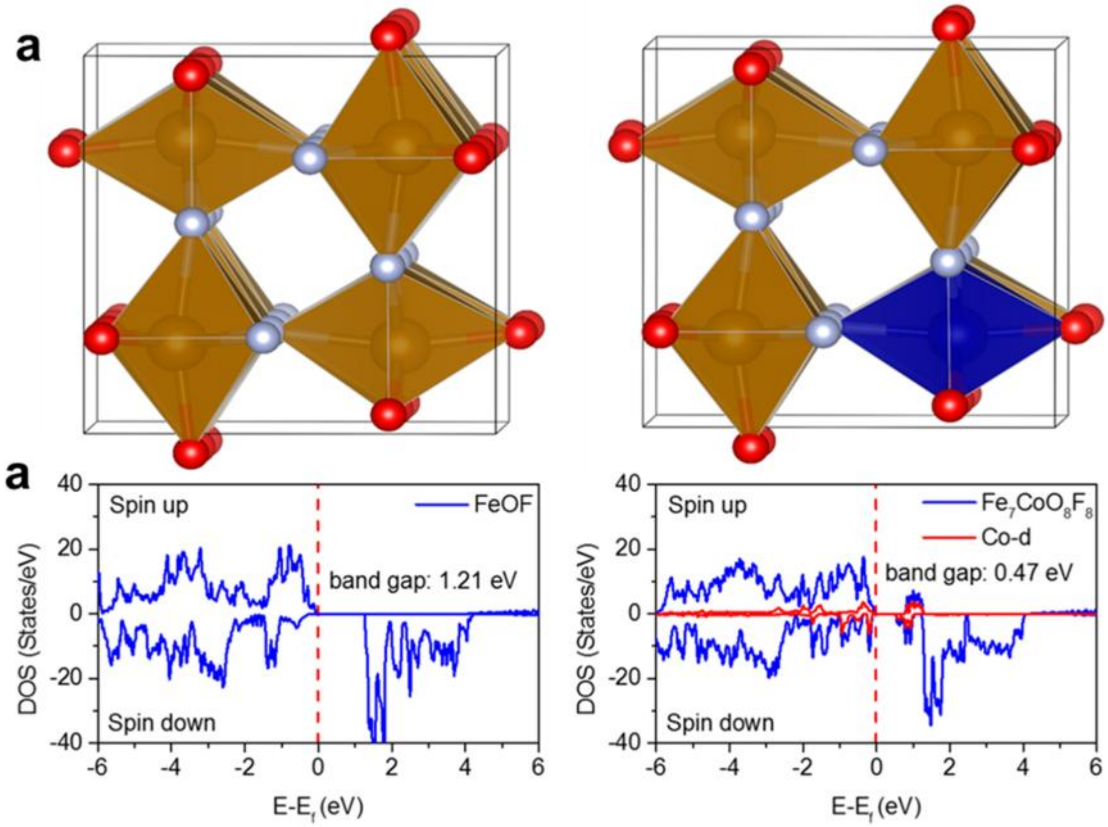

Supplementary Figure 3 Structure and calculated density state of the materials. (a) Structure and (b) density of states of FeOF and $Fe_7CoO_8F_8$. We constructed the $Fe_7CoO_8F_8$ model to calculated the density of states. The band gaps of FeOF and $Fe_7CoO_8F_8$ are calculated to be 1.21 eV and 0.47 eV by GGA+U, respectively. Substitutional Co narrow down the band gap due to the Co-d conduction band.

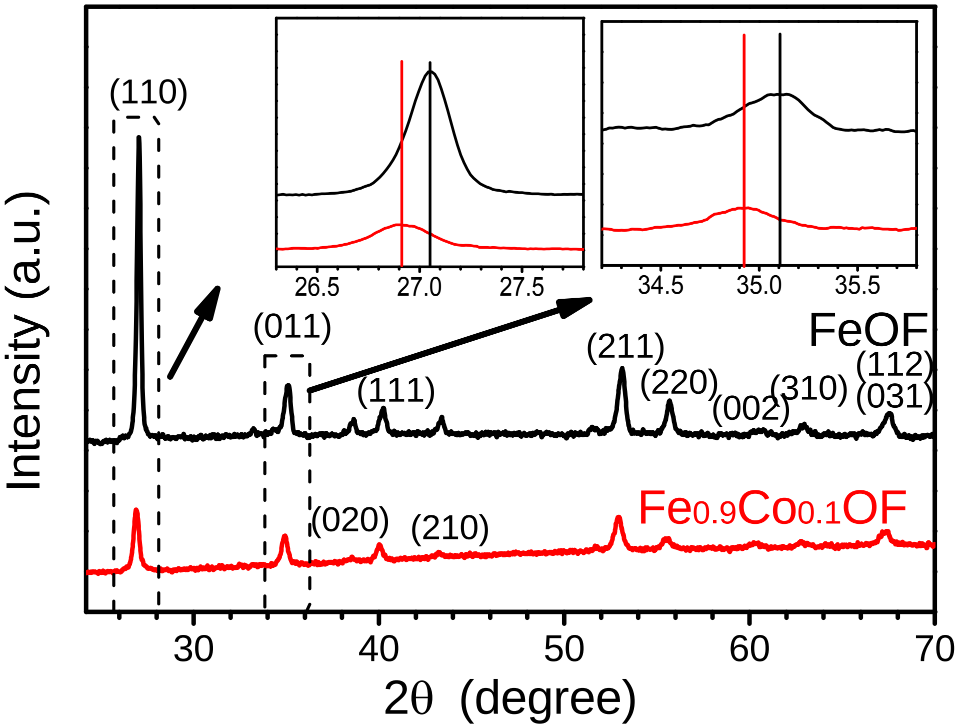

Supplementary Figure 4. XRD comparison for the as-synthesized $Fe_{0.9}Co_{0.1}OF$ and FeOF.

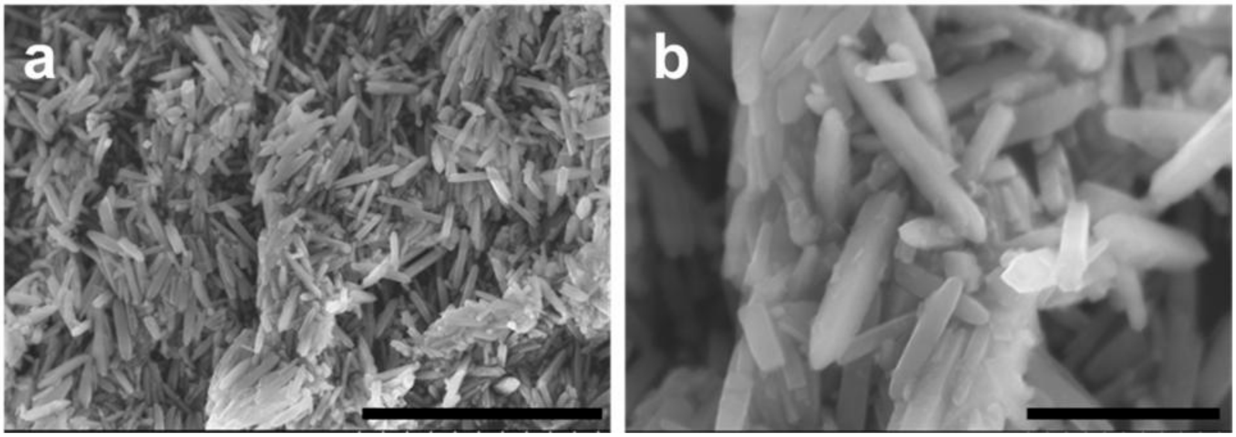

Supplementary Figure 5. SEM images of the FeOF synthesized in 1-proponal at 210 °C. Scale bar in a and b is 5 μm and 1 μm, respectively.

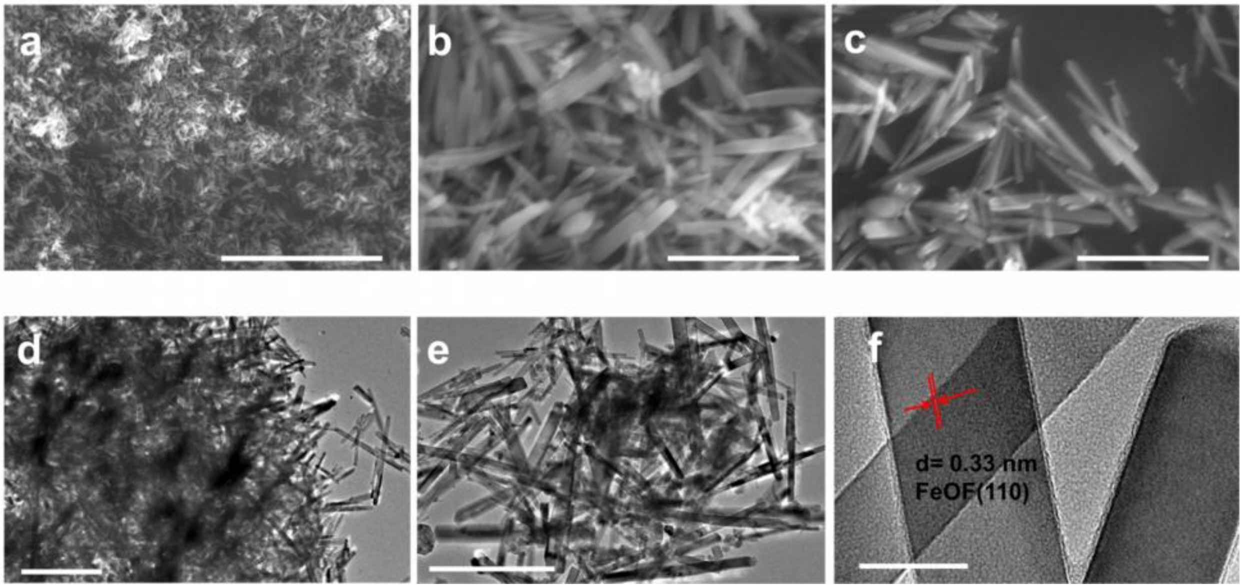

Supplementary Figure 6. Microstructures of the FeOF material. SEM (a, b, and c), TEM (d and e), and HRTEM (f) images for the as-synthesized FeOF material synthesized in Butyl-alcohol at 200 °C. It should be pointed out that the particle size of the as-synthesized FeOF is correlated with the solvents and temperatures utilized during synthesis. If 1-proponal is used as the solvent at 210 °C, the particle size of the FeOF is about 200nm × 1 µm, as shown in Figure S5. As the solvent changed to Butyl-alcohol at a temperature of 200 °C, the particle size of the as-synthesized FeOF has a diameter of 40-50 nm and length of 300-400 nm, which is similar to the $Fe_{0.9}Co_{0.1}OF$ rods. Scale bar in a is 5 µm. Scale bar in b, c, d, and e is 500 nm. Scale bar in f is 20 nm.

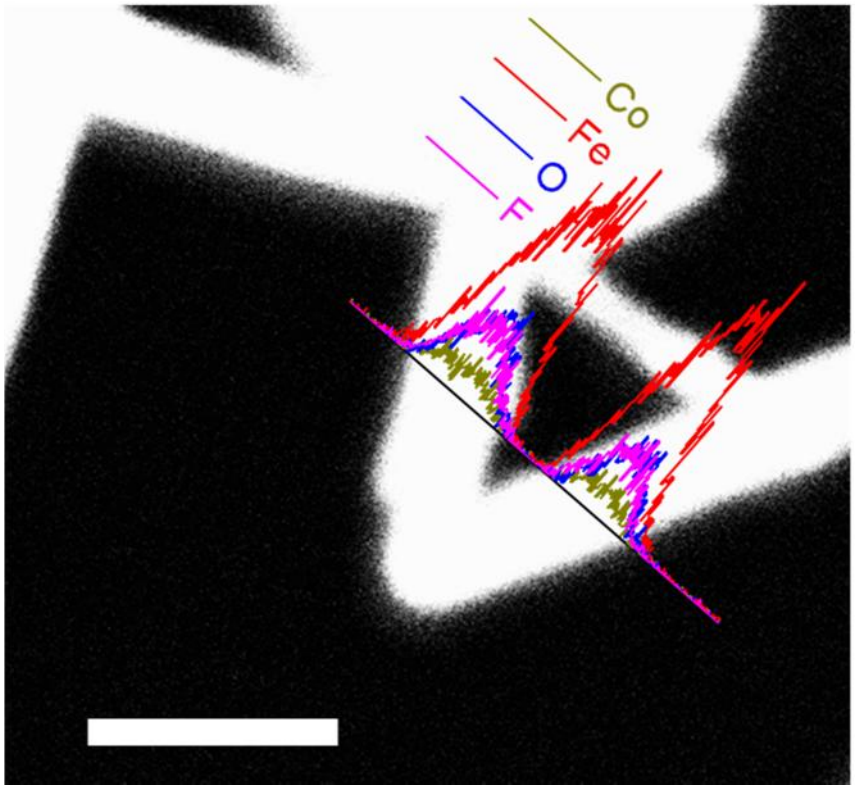

Supplementary Figure 7 STEM-HAADF image of the as-synthesized $Fe_{0.9}Co_{0.1}OF$ and the STEM-EDS line profiles for Fe, Co, O, and F elements. Scale bar is 100 nm.

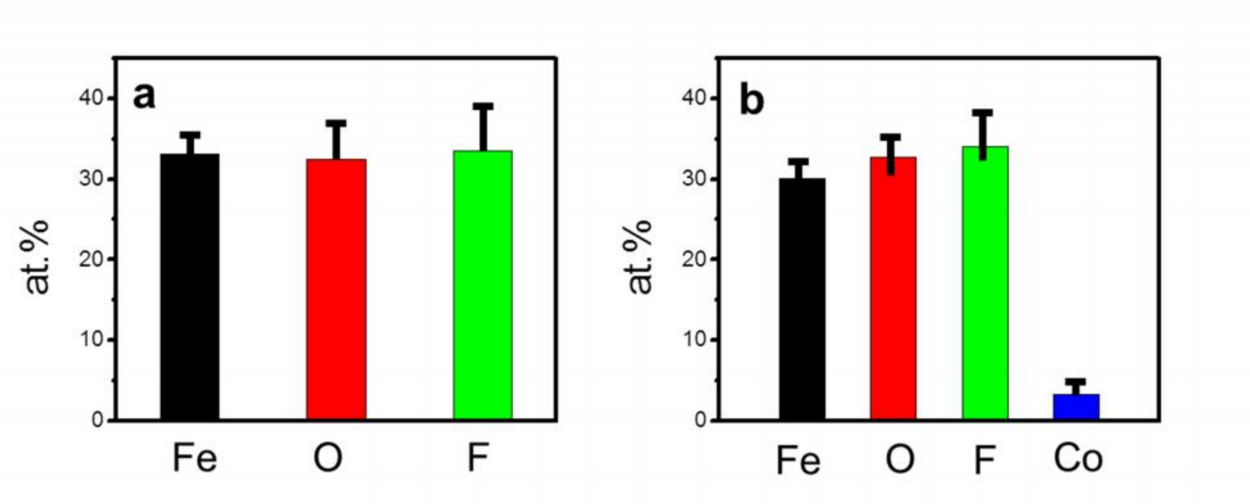

Supplementary Figure 8. Atomic ratio of different elements in FeOF (a) and $Fe_{0.9}Co_{0.1}OF$ (b). To get the detailed different element content in the two different samples, we characterized the FeOF and $Fe_{0.9}Co_{0.1}OF$ samples using energy dispersive spectroscopy (EDS). To be accurate, we characterized 20 different particles for these two samples. It can be seen that both of the FeOF (Fe: O: F = 33.1: 32.4: 33.5) and $Fe_{0.9}Co_{0.1}OF$ (Fe: Co: O: F = 30.1: 3.2: 32.7: 34) samples possess the similar O contents.

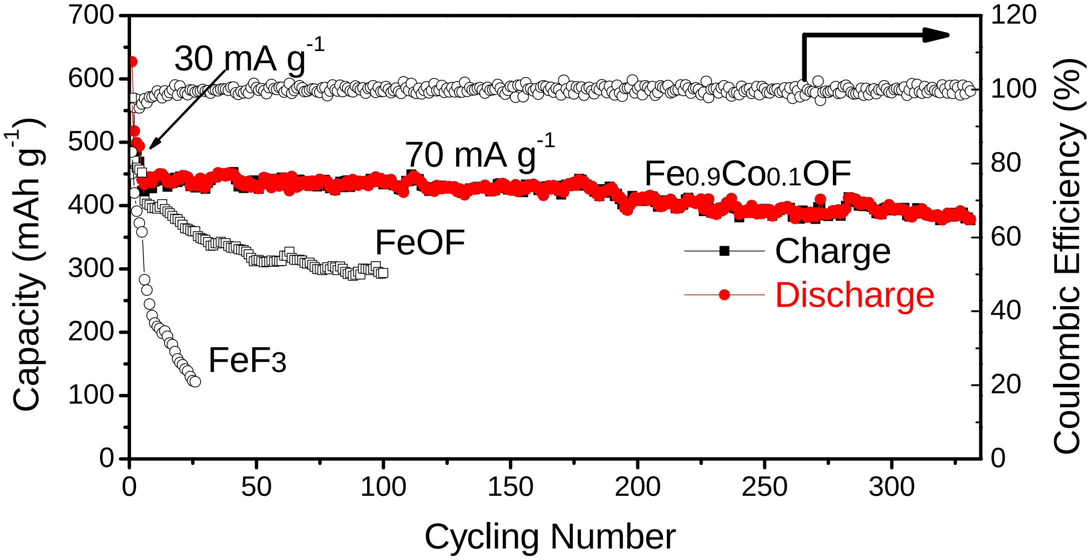

Supplementary Figure 9. The cycling performance of $Fe_{0.9}Co_{0.1}OF$, FeOF, and FeF_3 at a current of 70 mA g^{-1}.

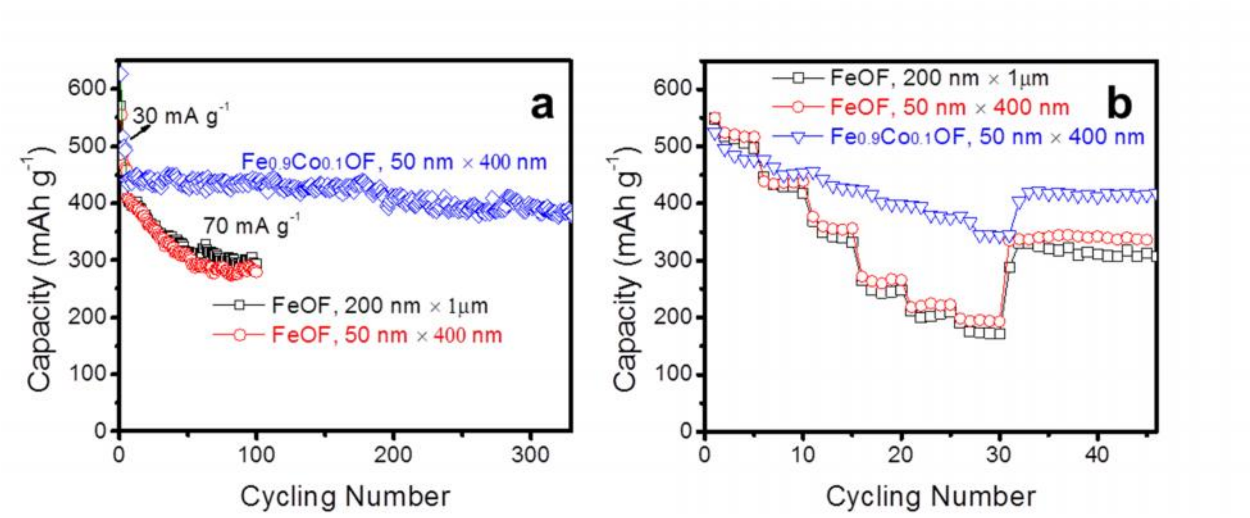

Supplementary Figure 10 Cycling performance and rate capability for the different sized FeOF materials. The related SEM images and the TEM images for the two different sized FeOF are shown in Supplementary Figure 5 and Supplementary Figure 6. The cycling performance and rate capability for the $Fe_{0.9}Co_{0.1}OF$ are also demonstrated.

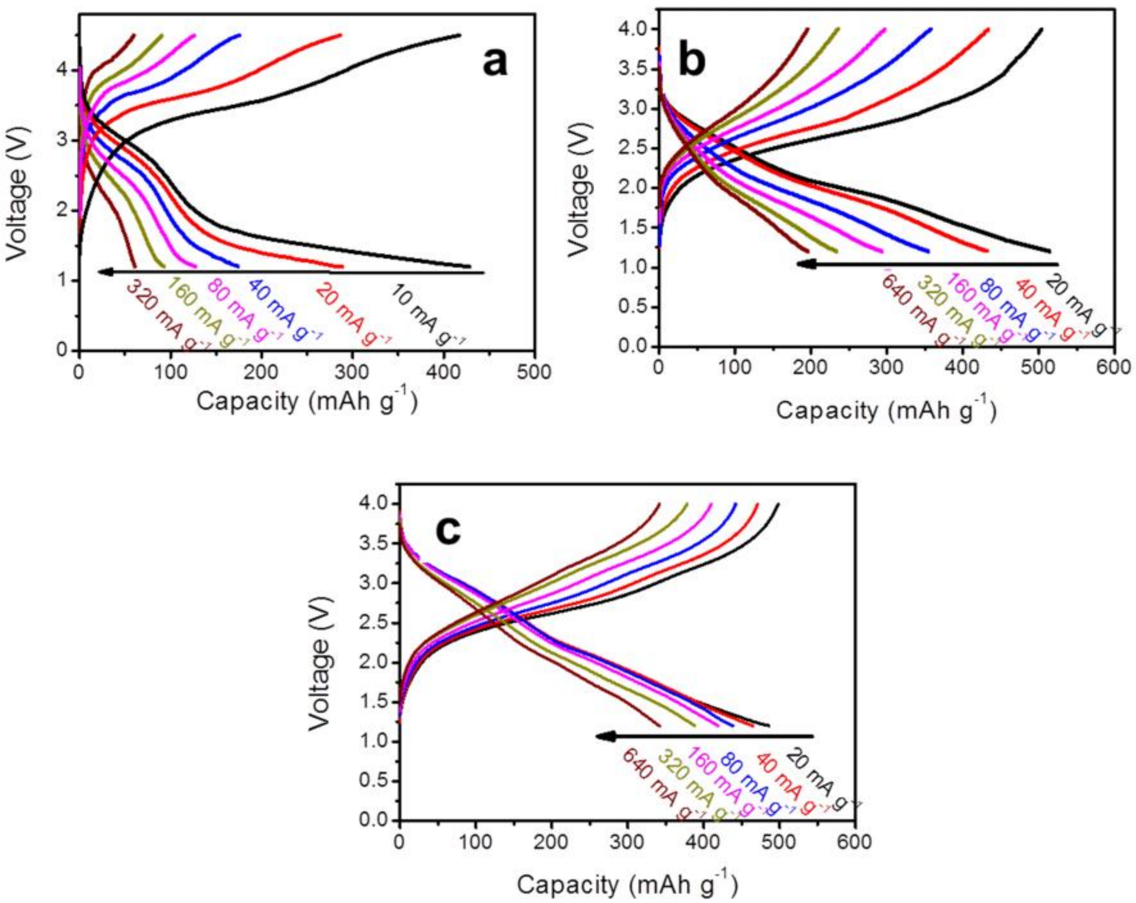

Supplementary Figure 11 Charge/discharge profiles at different currents for the different cathode materials. (a) the ball milled FeF_3/C composite; (b) as-synthesized FeOF nanorods and (c) $Fe_{0.9}Co_{0.1}OF$ nanorods. For FeF_3/C composite the charge/discharge voltage range is 1.2-4.5 V, while for FeOF and $Fe_{0.9}Co_{0.1}OF$ the range is 1.2-4.0 V. FeOF nanorods have similar size as $Fe_{0.9}Co_{0.1}OF$ nanorods. All cells have been activated at current density of 10 mA/g for two cycles.

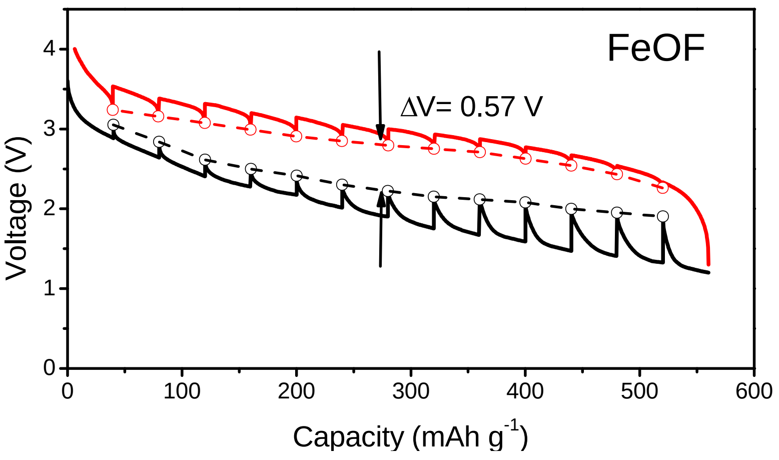

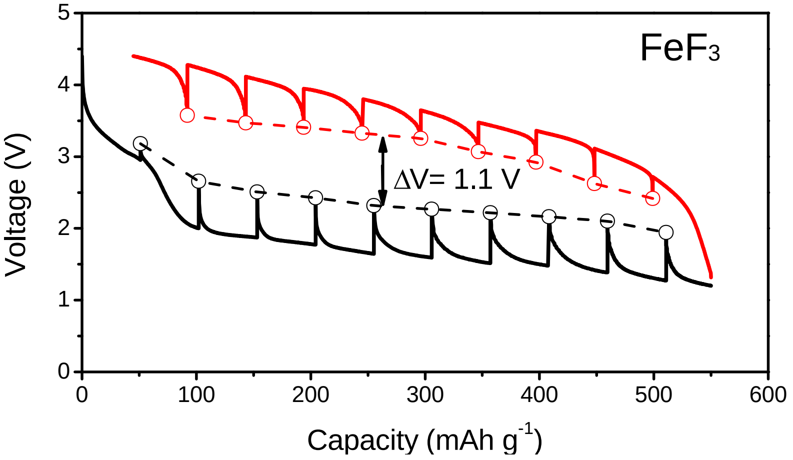

Supplementary Figure 12 Quasi-equilibrium voltage profile for FeOF and FeF_3 obtained from galvanostatic intermittent titration technique (GITT) measurements (for FeOF, 40 mA g^{-1} for 1 h followed by a 20 h rest; for FeF_3, 50 mA g^{-1} for 1 h followed by a 20 h rest). All electrochemical tests were conducted at room temperature.

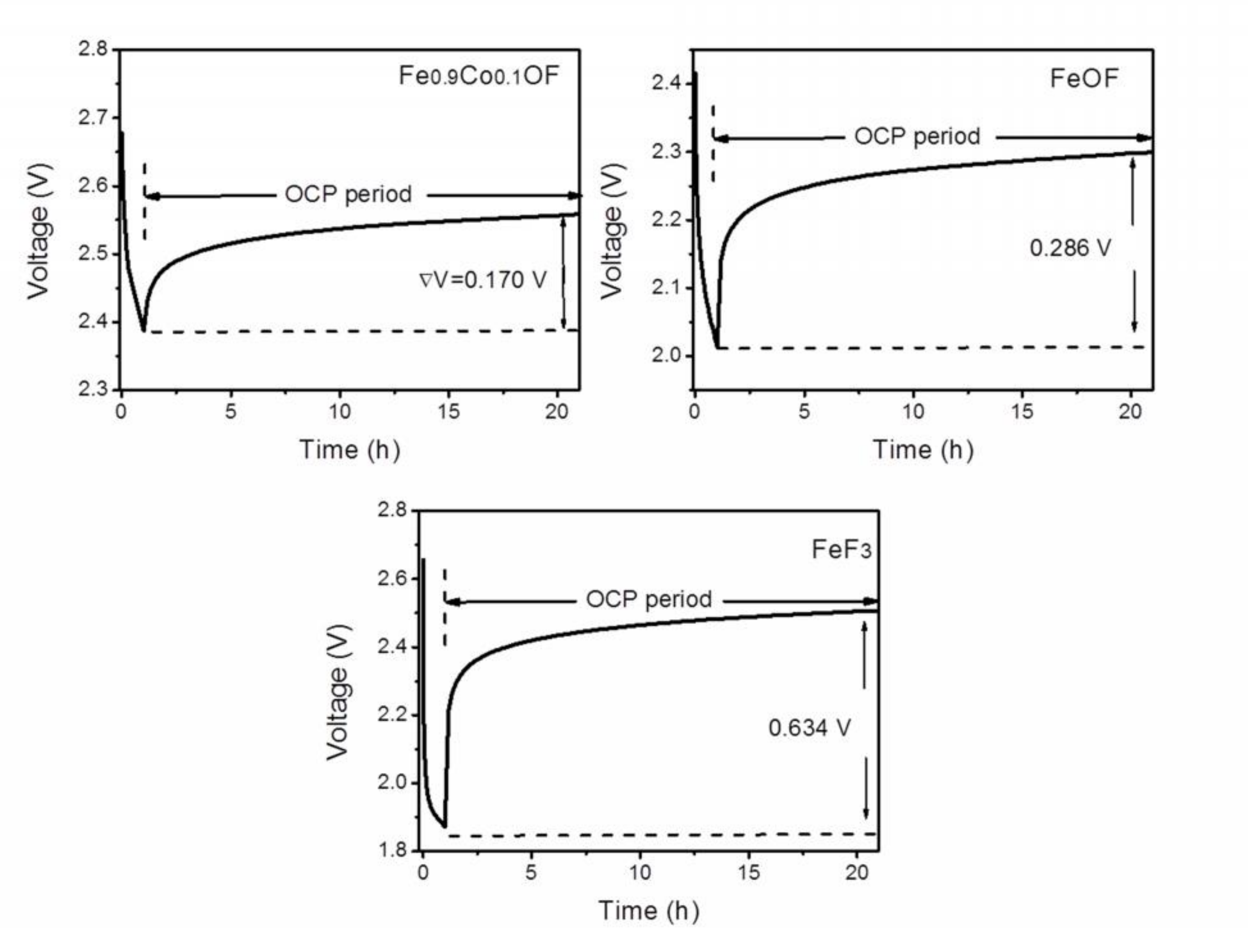

Supplementary Figure 13 Representative potential changes in lithiation and the relaxation process at open-circuit for $Fe_{0.9}Co_{0.1}OF$, FeOF and FeF_3 during GITT experiments. The electrodes are discharged for 1 h and rested for 20 h to reach equilibrium. All these segments represent the initial conversion reaction section for these three materials, which can be clearly seen from the GITT measurements (Figure 2f and Supplementary Figure 12).

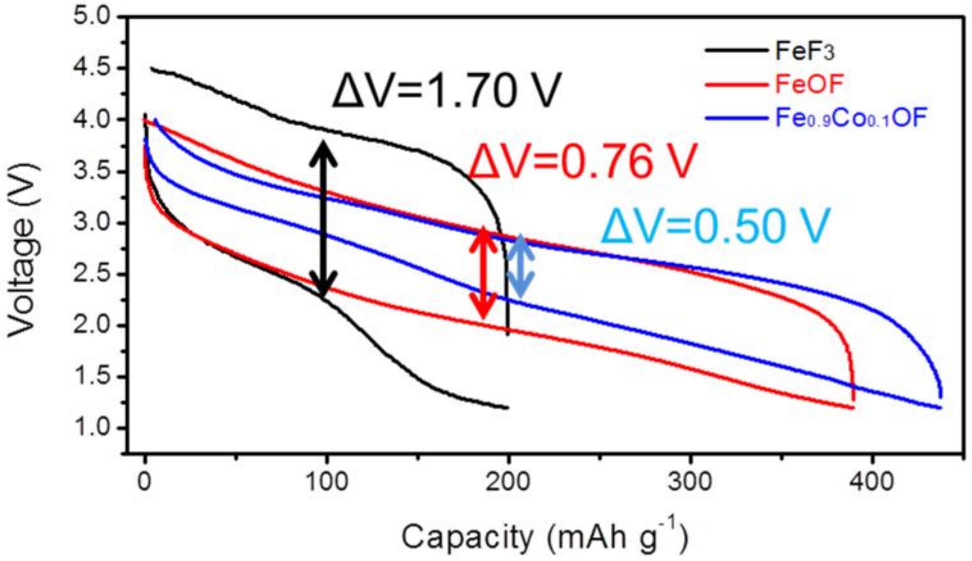

Supplementary Figure 14 charge/discharge profiles for FeF_3, FeOF, and $Fe_{0.9}Co_{0.1}OF$ cathode at 70 mA g^{-1}; The labeled over-potential value is calculated from the middle points of charge/discharge capacities for all three materials;

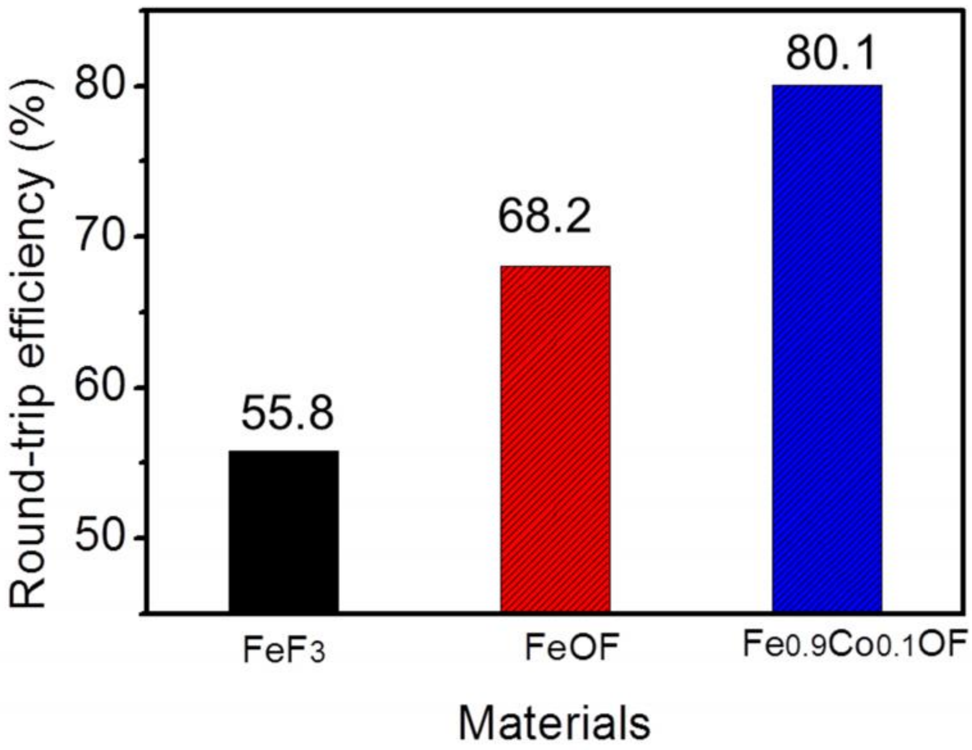

Supplementary Figure 15. Round-trip energy efficiency for three fluoride materials, whose calculation is based on a charge/discharge capacities and average voltages at 70 mA g^{-1}.

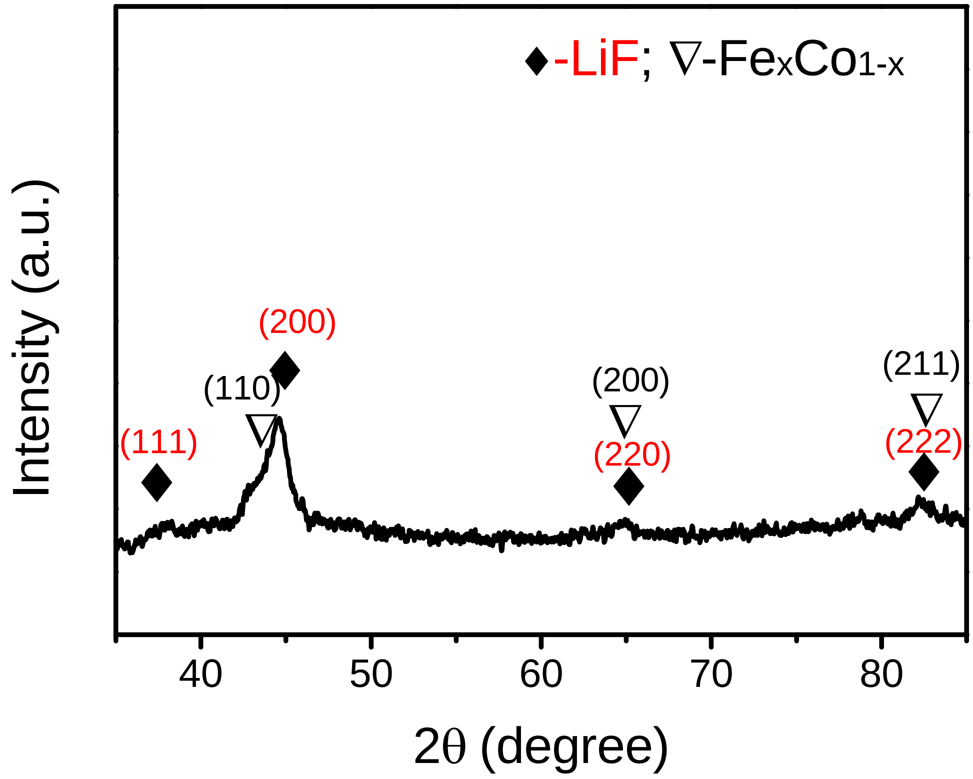

Supplementary Figure 16. XRD pattern for the pre-lithiated $Fe_{0.9}Co_{0.1}OF$ cathode material. The prelithiation reaction corresponds to $2LiH + Fe_{0.9}Co_{0.1}OF$ by ball milling. The possible product of Li-Fe-O compound is significantly distorted or becomes amorphous after *in situ* pre-lithiation during high-energy ball milling processes.

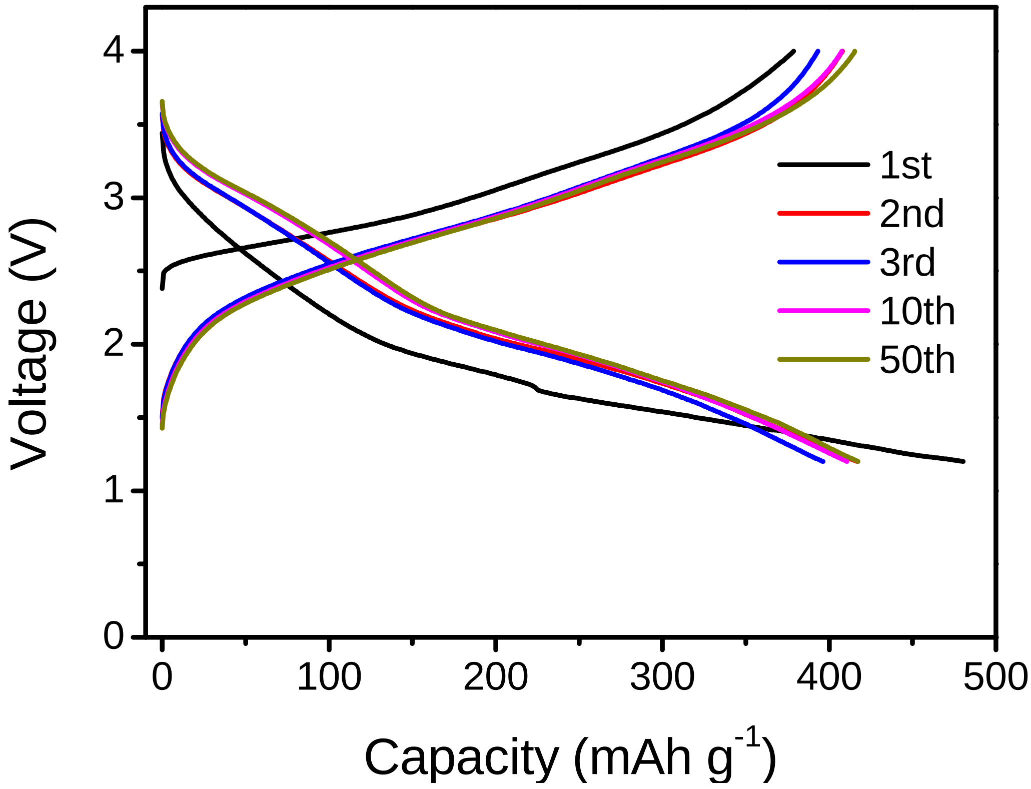

Supplementary Figure 17 charge/discharge curves for the pre-lithiated $Li_xFe_{0.9}Co_{0.1}OF$ cathode (100 mA g^{-1}).

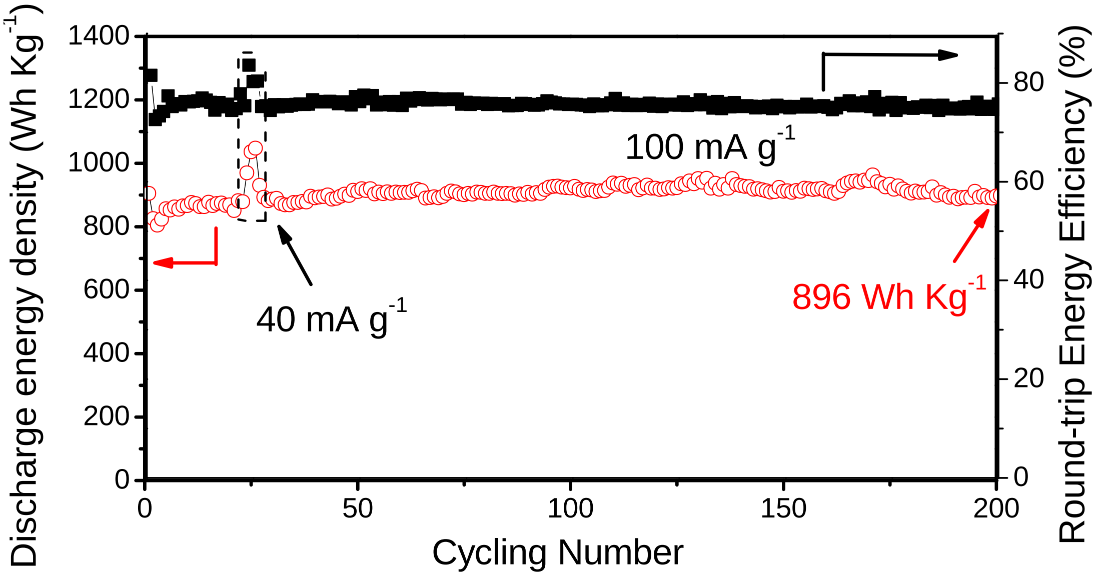

Supplementary Figure 18. Cycling performance for the pre-lithiated $Fe_{0.9}Co_{0.1}OF$ cathode.

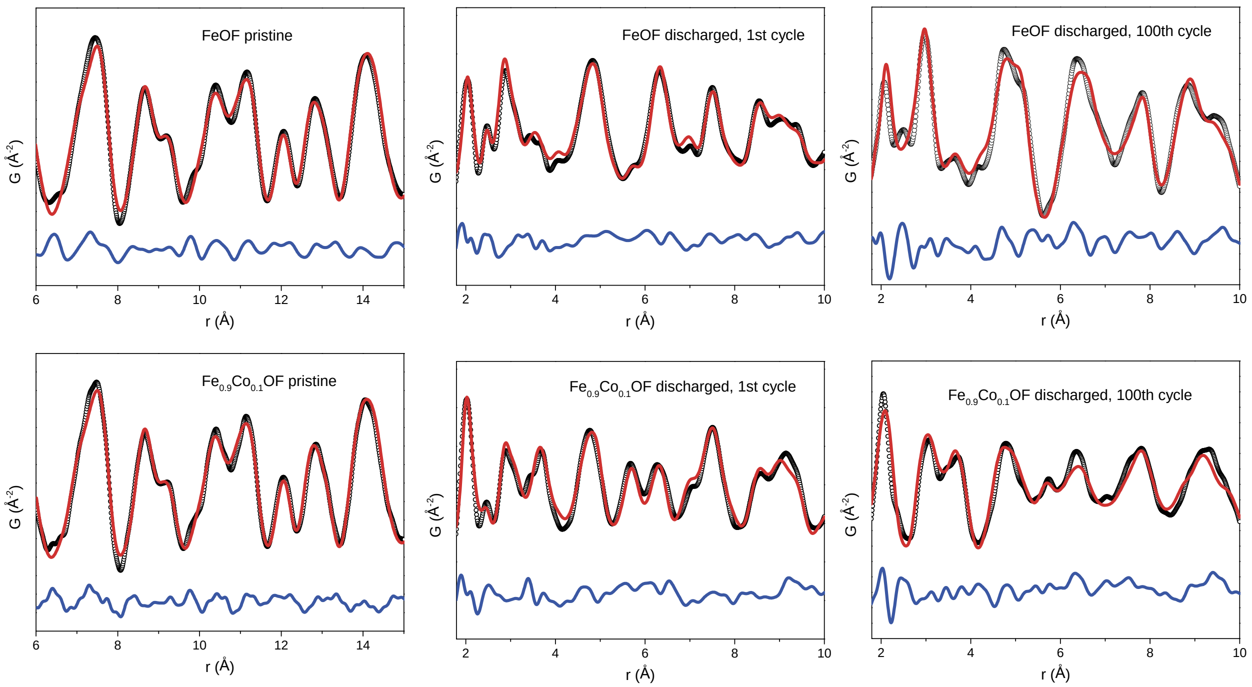

Supplementary Figure 19. PDF fitting results of FeOF and $Fe_{0.9}Co_{0.1}OF$ samples at different states.

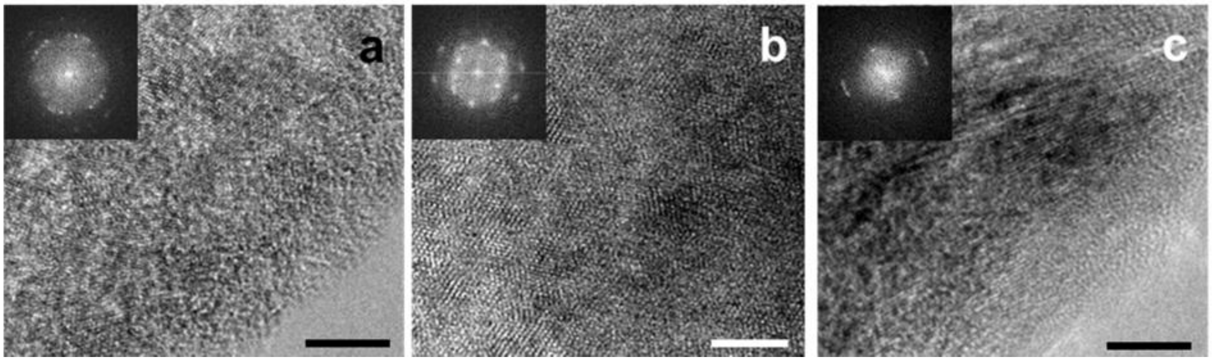

Supplementary Figure 20. HRTEM images and the corresponding fast Fourier transform (FFT) images for the $Fe_{0.9}Co_{0.1}OF$ samples: (a) discharged to 1.2 V; (b) charged to 4 V; (d) after 100 cycles. Scale bar in a, b, and c is 5 nm.

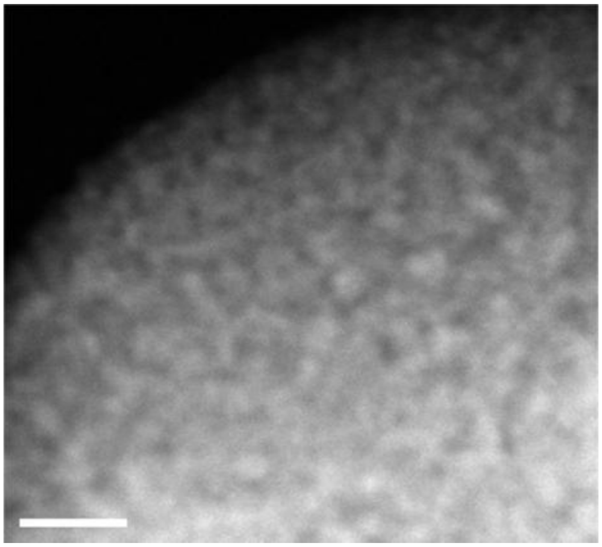

Supplementary Figure 21. STEM-HAADF image for FeOF after being discharged to 1.2 V. Scale bar is 10 nm.

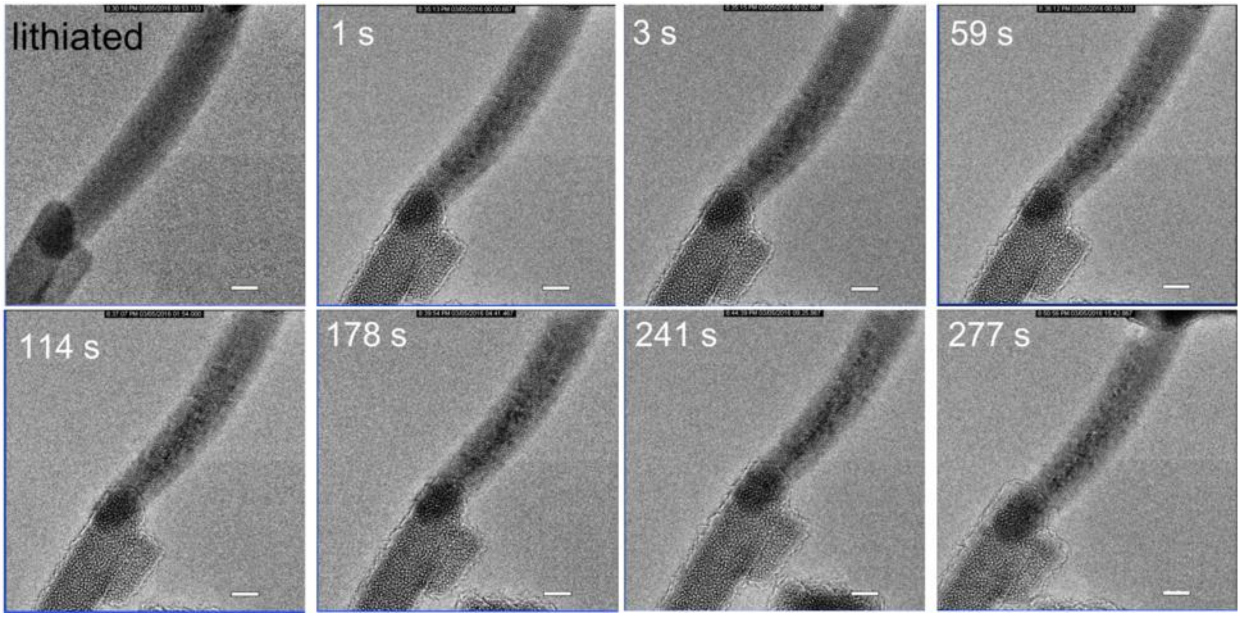

Supplementary Figure 22. Micro-morphological evolution of $Fe_{0.9}Co_{0.1}OF$ in delithiation process (1 s, 3 s, 59 s, 114 s, 178 s, 241 s, 277 s) and its comparison with lithiated (discharged) state. In contrast to the evident volume change during the first lithiation, the volume remains almost constant during cycling. Once voltage is applied, nanoparticles show up in the nanorods immediately (in < 3 seconds), then morphology and nanostructure of $Fe_{0.9}Co_{0.1}OF$ almost remain the same. This serves as a further proof for the ultra-fast delithiation reaction of the $Fe_{0.9}Co_{0.1}OF$ cathode. After the following delithiation (charge), the volume shrunk but still remain larger than the pristine material. This may be due to the largely reduction of crystal size for the cycled materials, while the pristine material is a single crystal. Lots of defects among the in-situ formed nano-crystals result in a slight volume increase for the regenerated $Fe_{0.9}Co_{0.1}OF$ compared with the pristine material. Scale bar is 20 nm

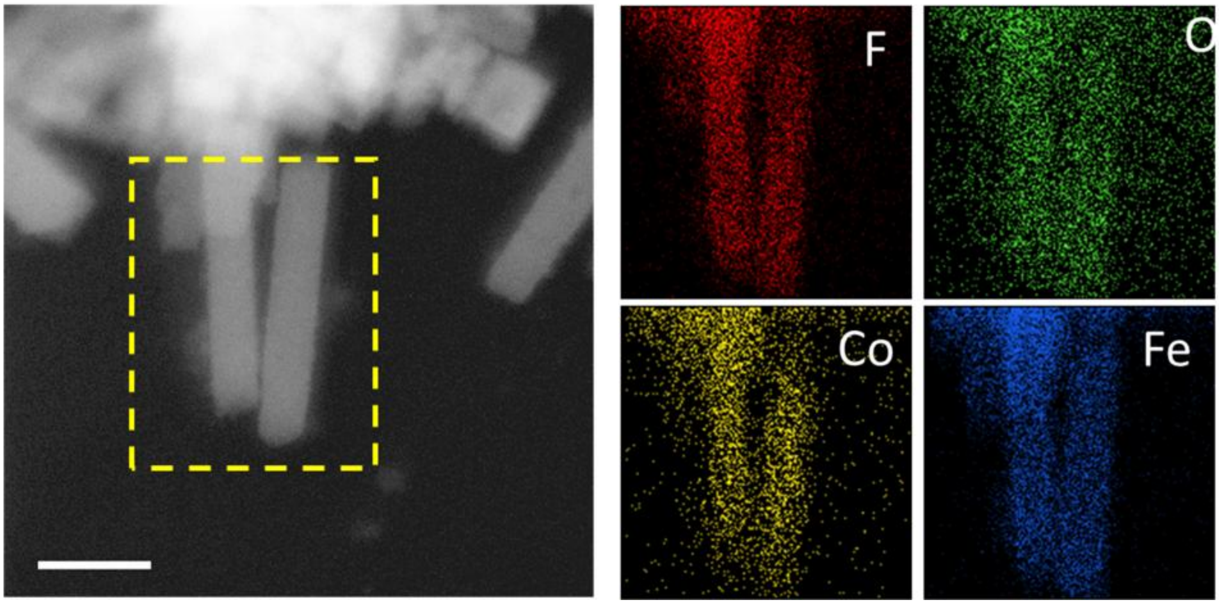

Supplementary Figure 23. STEM-EDS elemental mapping of F, O, Co, Fe, for $Fe_{0.9}Co_{0.1}OF$ after delithiation. Scale bar is 50 nm.

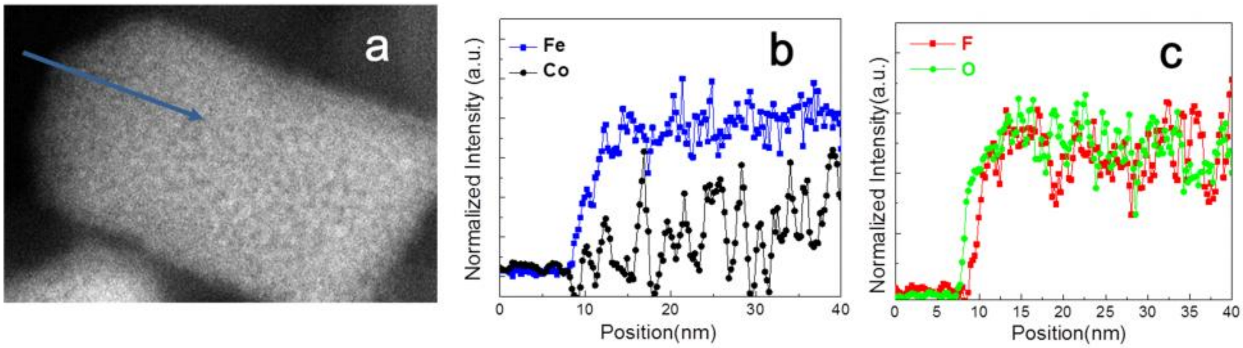

Supplementary Figure 24 The elemental distribution of $Fe_{0.9}Co_{0.1}OF$ after 100th lithiation. (a) ADF-STEM image, the cross-sectional EELS line profiles of Fe and Co (b), and O and F (c) after 100 cycles.

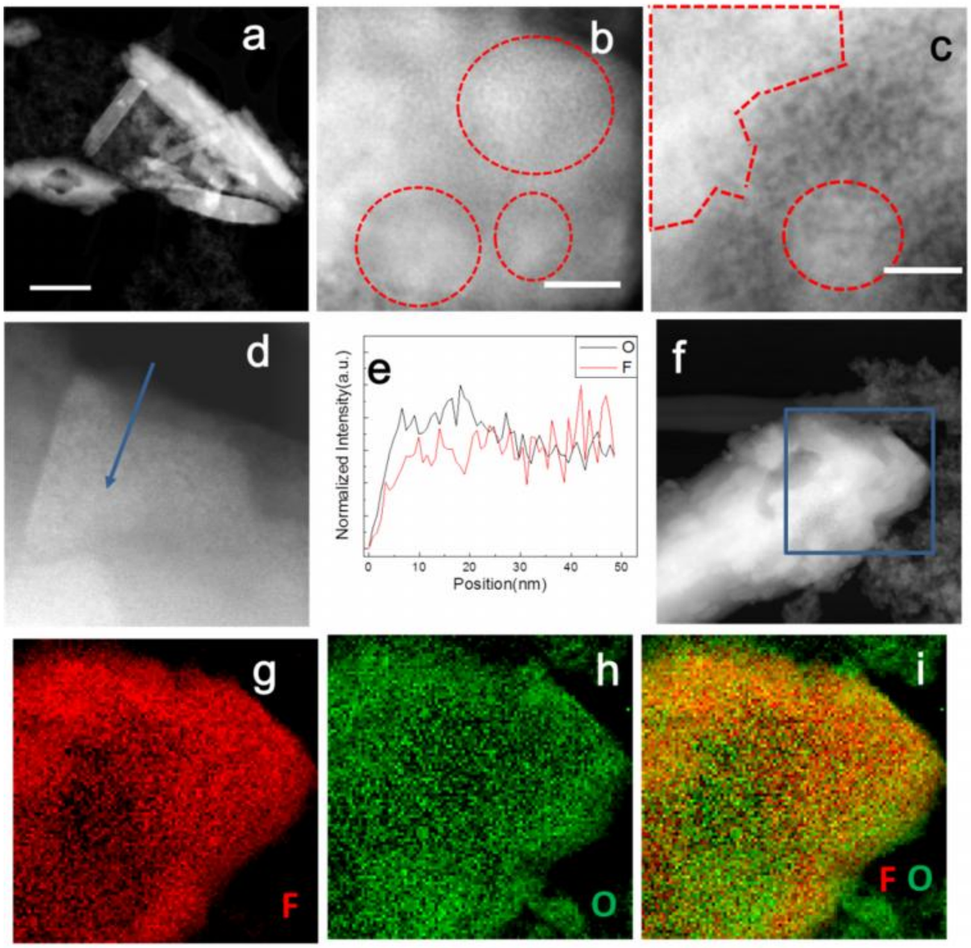

Supplementary Figure 25 Micro-morphology and structure evolvement after 100 charge/discharge cycles for FeOF. (a) (b) and (c) are STEM-HAADF images; (d) and (e) the cross-sectional compositional line-scan profiles of O, and F elements after 100 cycles; (f), (g), (h) and (i) the elemental mapping of O, F for FeOF after 100 cycle. Obvious nano-phase separation is observed, as denoted by the dash red lines in (b) and (c). The intensity of STEM-HAADF is proportional to $z^{1.7}$ (z is atomic number), therefore the circled areas should contain a high concentration of Fe, and the other areas should contain compositions of Li, F and O. The significant phase separation unavoidably results in poor reversibility and an obvious capacity decay as cycling proceeds. Besides, significant break-down was detected, as shown in Figure a and f, further deteriorating the reversibility. The distribution of F and O also shows obvious

enrichment in some areas (g, h, and i), in line with the phase separation observed in Figure b and c. All these phenomena constitute the reasons for the obvious capacity decay for FeOF cathode. Scale bar in a is 500 nm. Scale bar in b and c is 20 nm.

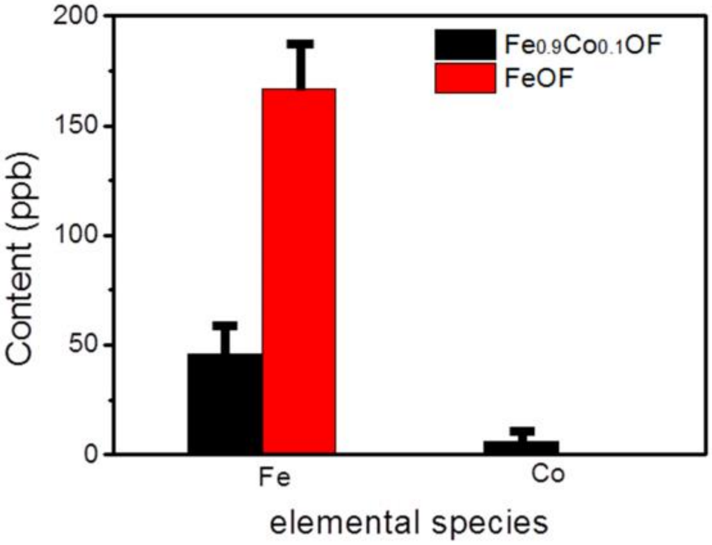

Supplementary Figure 26. ICP-AES (Inductively coupled plasma atomic emission spectroscopy) analysis of the cycled electrolytes recovered from cells based on $Fe_{0.9}Co_{0.1}OF$ and FeOF electrodes, respectively. For ICP-AES tests, 50 μL electrolytes were added into the coin cells, where the active materials for $Fe_{0.9}Co_{0.1}OF$ and FeOF are both 2 mg. After 100 cycles, the coin cells were disassembled and washed with 2 mL DMC and then dispersed in 10 mL HNO_3 aqueous solution for ICP-AES tests. To be accurate, we tested 8 samples for each samples.

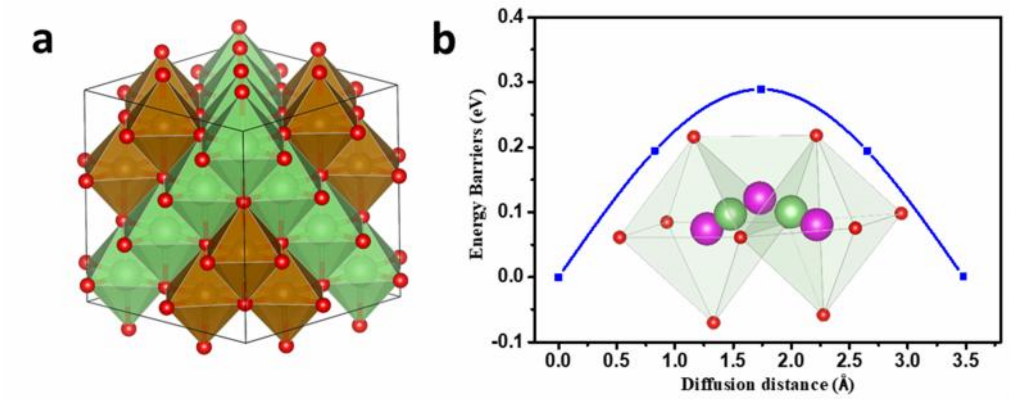

Supplementary Figure 27. Structure of the rocksalt $LiFeO_2$, and the corresponding Li diffusion barriers. (a) Illustration of rocksalt $LiFeO_2$, the ocher and green octahedral represent the FeO_6 and LiO_6 structure, respectively; (b) Calculated Li diffusion energy barriers and its corresponding diffusion path in $LiFeO_2$ using CINEB method.

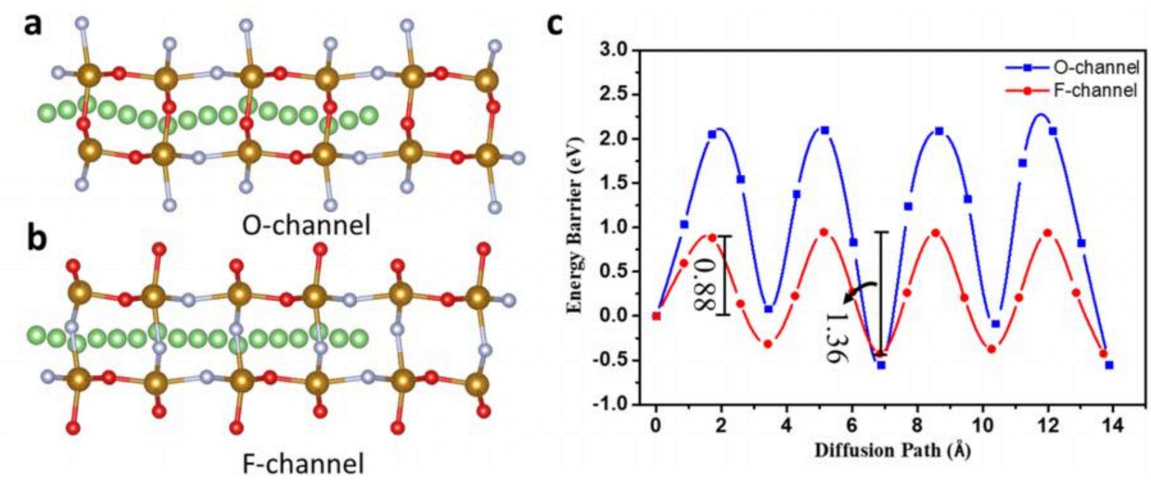

Supplementary Figure 28. Considered diffusion path from the surface to the bulk via (a) the O-channel (b) F-channel and (c) their calculated relative energies.

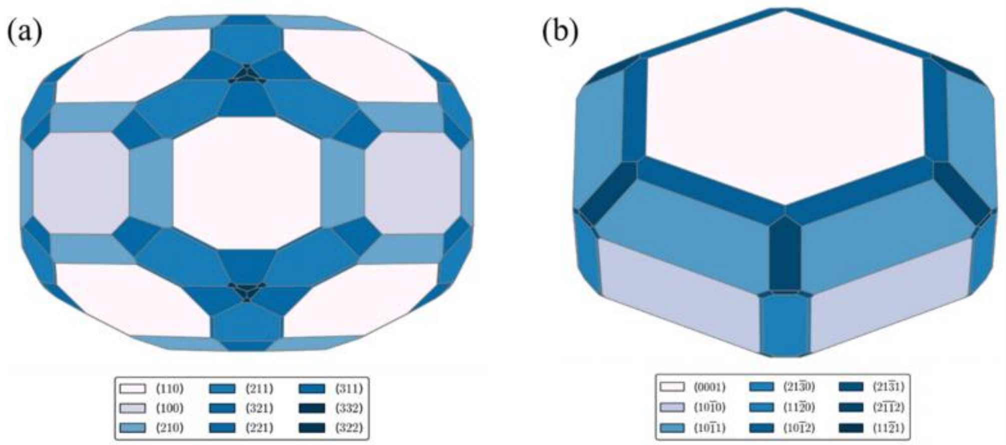

Supplementary Figure 29. Constructed Wulff shapes of different metals under equilibrium condition. (a) Fe and (b) Co.The weighted surface energies used in this work are 0.158 and 0.146 eV Å^{-2}, respectively. Pymatgen is used to generate shapes and calculate weighted surface energy.[1]

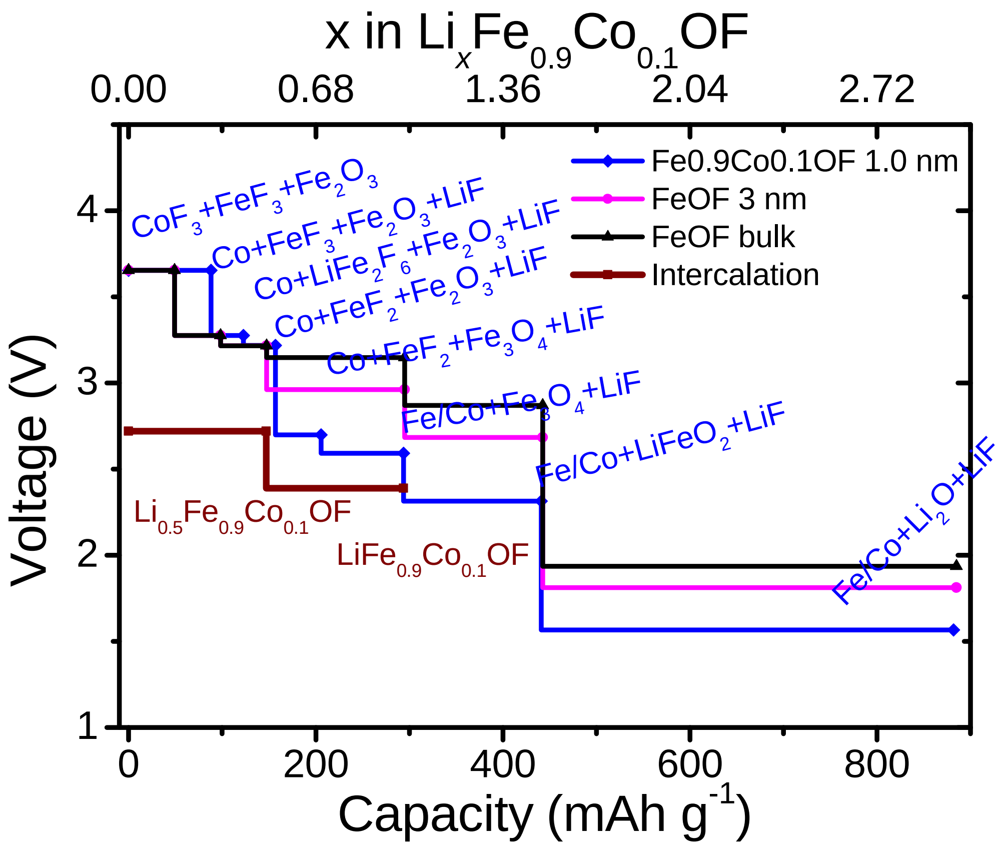

Supplementary Figure 30. Comparison of calculation reaction curves of the $Fe_{0.9}Co_{0.1}OF$ and FeOF materials from the DFT intercalation, and the conversion path using the equilibrium phases

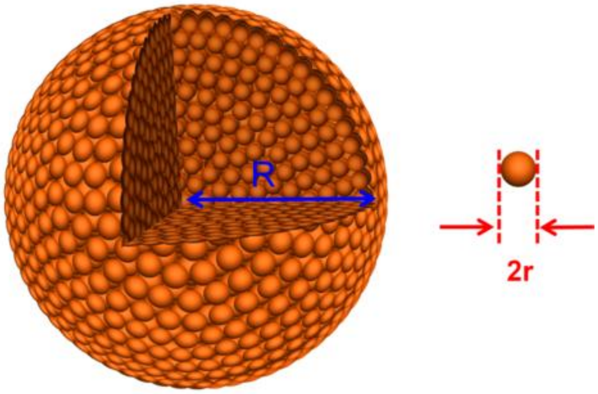

Supplementary Figure 31 Representative Fe nanoparticles and Fe atoms, assuming the *in situ* formed Fe nanoparticles are spherical.

Supplementary Table 1. The calculated surfaces with different Miller index and terminals and their corresponding surface energies.

**Surface**	**100**		**110**		
	1	2	1	2	3
**Surface energy (J m^{-2})**	0.55	0.28	0.94	0.55	0.82

Supplementary Note 1. Analysis of Li diffusion in the surface thin layer of O-rich rocksalt phase

The layer with thickness of ~ 2 nm will not inhibit the Li insertion in later cycles according to our DFT energy barrier calculation, as shown in Supplementary Figure 27. The main component for the oxide surface layer is considered to be rocksalt $LiFeO_2$ according to our results shown in the manuscript. To study the Li diffusion in the $LiFeO_2$, the energy barrier is calculated by climbing image nudged elastic band (CINEB) method implemented in VASP.[2] Other computation details remain the same as we used in the manuscript. According to the symmetry of $LiFeO_2$, only one diffusion path is considered. The calculated diffusion energy barriers and its corresponding diffusion path are plotted in Supplementary Figure 27. According to the results, Li diffuses from the octahedral to the tetrahedral site where the transition state lies on. The diffusion barrier is calculated to be 0.29 eV. This diffusion barrier is similar to that in $LiFePO_4$, which is considered to be a high rate cathode.[3]

Supplementary Note 2. Analysis of Li migration barrier in the $Fe_{0.9}Co_{0.1}OF$

To assess the surface to bulk migration barrier, the (001) surface terminated by F layer and having a vacuum of 12 Å is used as it is calculated to have the lowest energy. The relative energy of Li ion migration from the surface to the bulk-like slab center via O-channel and F-channel are calculated, as shown in Supplementary Figure 28a, b. Constrained minimization is used to hold the depth of the Li ion fixed while all other degrees of freedom are relaxed, as the previous publication.[4] The results are shown in Supplementary Figure 28c. It can be found that F-channel is more suitable for the Li migration. The diffusion barrier at the surface is about 0.88 eV. However, the diffusion energy barrier for the bulk is calculated to be 1.36 eV which is higher than the surface diffusion barrier. Therefore, the surface energy barrier effect is not a key problem for the FeCoOF system.

Supplementary Note 3. Analysis for the surface energy barrier for the the $Fe_{0.9}Co_{0.1}OF$

A high surface energy barrier is found in many other cathode materials. However, the surface energy barrier effect is not obvious in our FeCoOF system according to the DFT calculation results. To investigate the effect, the slab structure of FeCoOF with a max Miller index of 1 is generated by Pymatgen.[5,6] According to the Task's classification,[7] only the stoichiometric surfaces with a vanishing dipole in the direction of the surface normal is considered in our calculation as listed in Supplementary Table 1. To calculate the surface energy, we use slabs more than 15 Å and a 12 Å vacuum is used for each model. The outmost five atom layers of each surface of the slabs are relaxed while the middle layers are fixed to model as bulk. The most energy favorable surface is (100) surface with F as the terminals, as shown in Supplementary Figure 28.

Supplementary Note 4. The ratio of the surface atoms for the Fe/FeCo nanoparticles

The ratio of surface atoms can be calculated by the following equation:

$$\text{Ratio} = \frac{4\pi(R-r)^2 \times {}^4/_3 r}{{}^4/_3 \pi R^3} \times 100\%$$

r_{Fe} = 0.17 nm

For iron fluoride, R=~ 2.5 nm, Ratio = 23 %;

For FeOF, R= ~ 1.5 nm, Ratio = 35.6 %;

For $Fe_{0.9}Co_{0.1}OF$, R = 0.9 nm, Ratio = 49.3 %

Supplementary Note 5. The relationship between the Fe particle size and the re-conversion rate

After lithiation of FeF_3, FeOF, and $Fe_{0.9}Co_{0.1}OF$, Fe or $FeCo_y$ nanoparticles with different particle size are dispersed in the LiF and Li-Fe-O matrix. Assuming the *in situ* formed Fe (or $FeCo_x$) alloy is spheres, the quantity Q of Fe present during re-conversion process at any time *t* is:

$$Q_{Fe} = \frac{4}{3}\pi r^3 \quad (1)$$

Wang et al showed that fluorine mobility within the rutile-like structure is lower than that of Li or Fe,[8] therefore the conversion/reconversion reaction is mainly controlled by the Li and Fe redistribution in different nanophases. Herein, the rate of change for Fe should be equal to the flux of Fe diffusing through the spherical shell of thickness *y*. The rate of thickening of the reconversion product (FeO_xF_y or Fe-Co-O-F) can be assumed inversely proportional to its thickness:

$$\frac{dy}{dt} = k/y \quad (2)$$

Integration of (2) gave:

$$y^2 = 2kt \quad (3)$$

The volume for the unreacted Fe nanoparticles at time t can be expressed as:

$$V = \frac{4\pi}{3}(r - y)^3 \quad (4)$$

Or $$V = \frac{4\pi}{3}r^3(1 - x) \quad (5)$$

where x is the fraction of the original sphere which has reacted. From Supplementary Equations (4) and (5), we can get:

$$y = r\left[1 - (1 - x)^{\frac{1}{3}}\right] \quad (6)$$

By substituting Supplementary Equation (6) into (3), we can get:

$$\left[1 - (1 - x)^{\frac{1}{3}}\right]^2 = 2kt/r^2 \quad (7)$$

From Supplementary Equation (7), it can be easily obtained that the reaction rate is inverse to r^2 for different particles. It should be noted that, for this equation, we made a simplification in equating Supplementary Equation (4) and (5). These volumes are equal only when the volume of sphere consisting of the unreacted Fe (or FeCo) and the reaction product is equal to the initial volume of Fe (or FeCo). In other words, Supplementary Equation (7) applies only at start of the reaction. As the reaction is nearing completion, this equation will deviate from the real value. As shown in Figure 4h, the particle size for the in situ formed FeCo nanoparticles is only about 1.8 nm compared with 3.0 nm for Fe nanoparticles. So according to Supplementary Equation (7), the re-conversion reaction rate for $Fe_{0.9}Co_{0.1}OF$ should be 3 times higher than that of FeOF at the beginning of the reconversion reaction, which are in good agreement with the superior rate capability for $Fe_{0.9}Co_{0.1}OF$.

Supplementary References

1. Tran, R. *et al.* Surface energies of elemental crystals. *Sci. Data* **3**, 160080 (2016).

2. Henkelman, G., Uberuaga, B. P. & Jónsson, H. A climbing image nudged elastic band method for finding saddle points and minimum energy paths. *J. Chem. Phys.* **113**, 9901-9904 (2000).

3. Dathar, G. K. P., Sheppard, D., Stevenson, K. J. & Henkelman, G. Calculations of Li-ion diffusion in olivine phosphates. *Chem. Mater.* **23**, 4032-4037 (2011).

4. Tompsett, D. A., Parker, S. C., Bruce, P. G. & Islam, M. S. Nanostructuring of β-MnO_2: the important role of surface to bulk ion migration. *Chem. Mater.* **25**, 536-541 (2013).

5. Sun, W. & Ceder, G. Efficient creation and convergence of surface slabs. *Surf. Sci.* **617**, 53-59 (2013).

6. Kamrani Moghaddam, L., Ramezani Paschepari, S., Zaimy, M. A., Abdalaian, A. & Jebali, A. The inhibition of epidermal growth factor receptor signaling by hexagonal selenium nanoparticles modified by SiRNA. *Cancer gene ther*. **23**, 321-325 (2016).

7. Tasker, P. The stability of ionic crystal surfaces. *J. Phys. C: Solid State Phys.* **12**, 4977 (1979).

8. Wang, F. *et al.* Tracking lithium transport and electrochemical reactions in nanoparticles. *Nat. Commun.* **3**, 1201 (2012).
