# Supplementary material for: High energy-density and reversibility of iron fluoride cathode enabled via an intercalation-extrusion reaction
Source: Nat Commun. 2018 Jun 13;9:2324. doi: 10.1038/s41467-018-04476-2 (PMC5998086; doi:10.1038/s41467-018-04476-2)
Supplement: Supplementary file 3 — Description of Additional Supplementary Files [file 41467_2018_4476_MOESM3_ESM.pdf]

Description of Additional Supplementary Files

File Name: Supplementary Movie 1
Description: In-situ TEM imaging of $Fe_{0.9}Co_{0.1}OF$ nanorod for their morphological evolution upon electrochemical lithiation.

File Name: Supplementary Movie 2
Description: In-situ TEM imaging of $Fe_{0.9}Co_{0.1}OF$ nanorod for their morphological evolution upon electrochemical delithiation.
